# Association of Mineralocorticoid Receptor Antagonists With the Mortality and Cardiovascular Effects in Dialysis Patients: A Meta-analysis

**DOI:** 10.3389/fphar.2022.823530

**Published:** 2022-05-17

**Authors:** Wen-Jun Gou, Fa-Wei Zhou, Rui Providencia, Bo Wang, Heng Zhang, Shou-Liang Hu, Xiao-Li Gao, Yan-hong Tuo, Yong Zhang, Tian Li

**Affiliations:** ^1^ Department of Nephrology, The First Affiliated Hospital of Yangtze University, Jingzhou, China; ^2^ Department of Emergency, The Central Hospital of Enshi Tujia and Miao Autonomous Prefecture, Enshi, China; ^3^ Barts Heart Centre, St. Bartholomew’s Hospital, London, United Kingdom; ^4^ Department of Ultrasound, The First Medical Center, Chinese People’s Liberation Army General Hospital, Beijing, China; ^5^ Department of Histology and Embryology, Xiang Ya School of Medicine, Central South University, Changsha, China; ^6^ Department of Nephrology, The Central Hospital of Wuhan, Tongji Medical College, Huazhong University of Science and Technology, Wuhan, China; ^7^ Department of Nephrology, Jianli People’s Hospital, Jingzhou, China; ^8^ School of Basic Medicine, Fourth Military Medical University, Xi’an, China

**Keywords:** hemodialysis, mineralocorticoid receptor antagonists, mortality, end-stage renal disease, cardiovascular mortaility

## Abstract

Whether Mineralocorticoid receptor antagonists (MRA) reduce mortality and cardiovascular effects of dialysis patients remains unclear. A meta-analysis was designed to investigate whether MRA reduce mortality and cardiovascular effects of dialysis patients, with a registration in INPLASY (INPLASY2020120143). The meta-analysis revealed that MRA significantly reduced all-cause mortality (ACM) and cardiovascular mortality (CVM). Patients receiving MRA presented improved left ventricular mass index (LVMI) and left ventricular ejection fraction (LVEF), decreased systolic blood pressure (SBP) and diastolic blood pressure (DBP). There was no significant difference in the serum potassium level between the MRA group and the placebo group. MRA vs. control exerts definite survival and cardiovascular benefits in dialysis patients, including reducing all-cause mortality and cardiovascular mortality, LVMI, and arterial blood pressure, and improving LVEF. In terms of safety, MRA did not increase serum potassium levels for dialysis patients with safety.

**Systematic Review Registration:** (https://inplasy.com/inplasy-protocol-1239-2/), identifier (INPLASY2020120143).

## Introduction

Worldwide, approximately 2 million people receive dialysis for end-stage renal disease (Caskey et al., 2011). End-stage renal disease is usually treated with hemodialysis or peritoneal dialysis (Song and Cai, 2017), however, the removal of electrolytes often induces capacity imbalance, which impairs cardiac function and increases cardiac load of patients, ultimately contributes to poor effects of dialysis treatment (Wang et al., 2018). Due to the influence of drugs, hypertension, biological incompatibility with dialysis and primary diseases, the residual renal function (RRF) of patients still decreases at a certain rate (Tian et al., 2016). Therefore, how to protect the RRF of patients is of great clinical significance for reducing mortality and cardiovascular complications of dialysis patients (Wang et al., 2018).

At present, angiotensin-converting enzyme inhibitors (ACEI) and angiotensin receptor blockers (ARB) are used in clinic to slow the maintain of RRF of patients. Although ACEIs are effective, the long-term application of these drugs may lead to aldosterone escape, and thus, they may not be able to completely inhibit the production of aldosterone (K/DOQI Workgroup, 2005; Shavit et al., 2012). As a result, the risk of cardiovascular adverse events is increased, and the progress of kidney disease is accelerated (Tian et al., 2016). MRA play a role by competitively inhibiting the binding of aldosterone ligands to receptors. Several studies (Zannad et al., 2000; Higgins and Deeks, 2011; Najafi et al., 2014) have confirmed that the long-term survival rate of patients with HFrEF and myocardial infarction can be significantly improved by the use of MRA during routine treatment (including ACEIs/ARBs). However, among patients undergoing maintenance dialysis, renal potassium excretion is reduced, and the risk of high blood potassium is very high (Ng et al., 2015). Notably, the risk/benefit ratio of MRAs in dialysis patients is less well defined, owing to concerns that their cardioprotective actions may be counteracted by excess risk of hyperkalemia (Georgianos et al., 2017). Moreover, patients with end-stage renal disease often experience multiple complications, consequently, mineralocorticoid receptor antagonists (MRA) should be considered for complete blockade of the renin-angiotensin-aldosterone system (RAAS) (Zhao et al., 2016). MRA, including eplerenone and spironolactone, have been shown effective in patients with hypertension and HFrEF (Pitt et al., 1999; Zannad et al., 2011). However, the use of MRA in patients undergoing hemodialysis remains controversial. MRA often leads to elevated serum potassium levels, thereby posing a risk of cardiac arrest (Pitt and Rossignol, 2014). A previous pooling analysis (Quach et al., 2016) showed that MRA was significantly associated with hyperkalemia, whereas some recently RCTs (Gross et al., 2005; Feniman-De-Stefano et al., 2015; Yongsiri et al., 2015; Charytan et al., 2019; Hammer et al., 2019) (randomized controlled trials) have revealed a different conclusion. The objective of this article is to evaluate the association of mineralocorticoid receptor antagonists with the mortality and cardiovascular effects in dialysis patients vs. placebo.

## Methods

### Study Protocol

This article is conducted in accordance to the Preferred Reporting Items for Systematic Reviews and Meta-Analyses (PRISMA) guideline (Liberati et al., 2009) and registered in INPLASY (DOI: 10.3776/inplasy2020.12.0143).

### Search Strategy

Two researchers (Zhang and Tuo) independently searched the PubMed, EMBASE and China National Knowledge Internet (CNKI) databases from inception to May 2020 by using medical subject headings (MeSH), Emtree, and text word with no language limitations.

The following keywords were used for search strategy: “mineralocorticoid receptor antagonists”, “MRA”, “spironolactone”, “eplerenone”, “haemodialysis”, “ESRD”, “dialysis” and “hemodialysis” (Supplementary Table S3). Reference lists from the identified studies were also searched for potentially eligible articles. Preliminary publications were imported into EndNote X9.1 (Clarivate Analytics, Philadelphia, United States), duplicate records and irrelevant literature were removed, and appropriate studies with detailed classification were compiled.

### Eligibility Criteria

**FIGURE 1:**
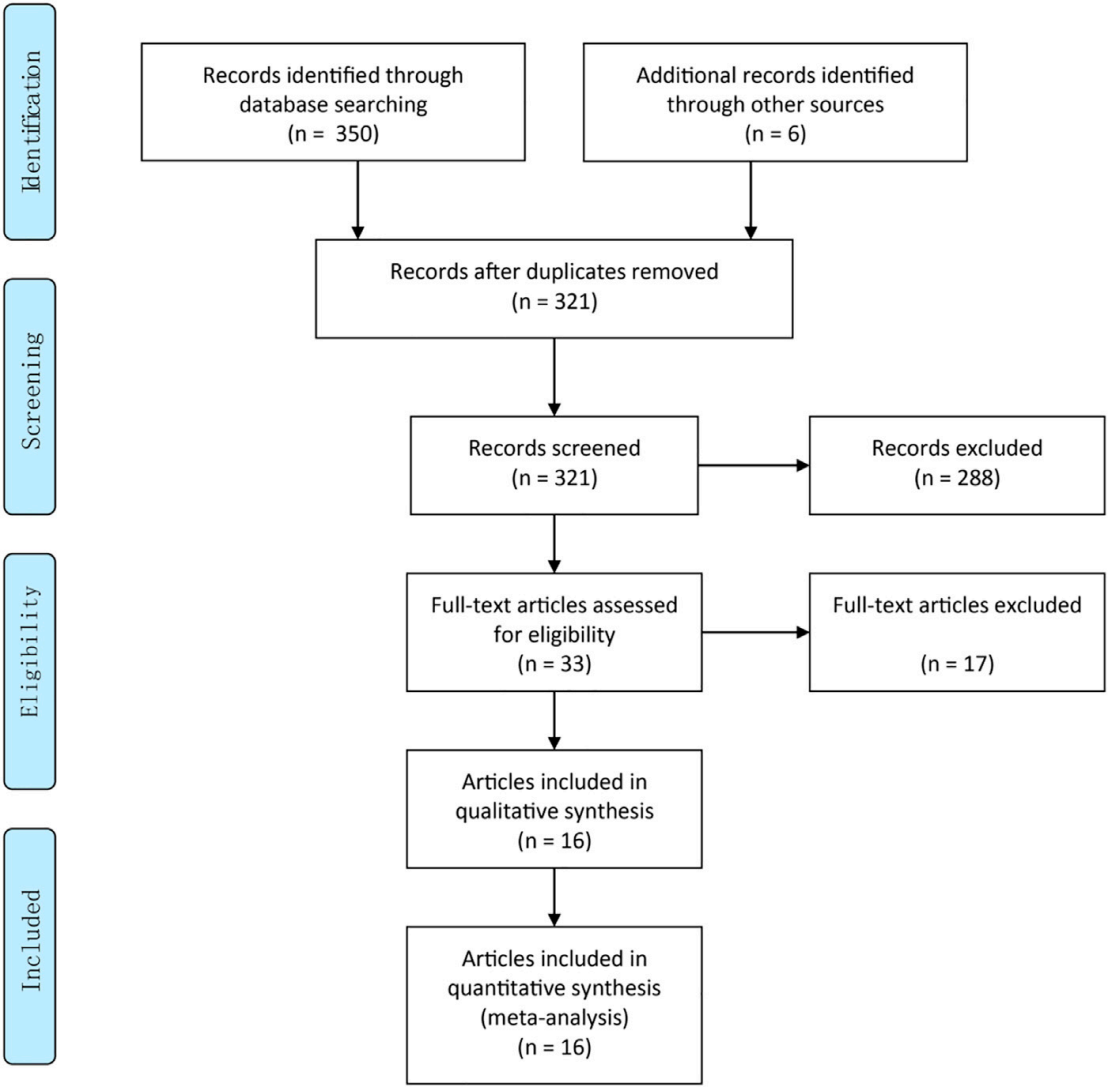
PRISMA 2009 flow diagram.

Two authors (ZY and Wb) independently carried out the primary review to search for trials that met the inclusion criteria (Supplementary Table S4. Any discrepancy was resolved by discussion and consensus (Figure 1). The following criteria were used: 1) Adults participants (≥18 y) were on dialysis for at least 1 month, irrespective of age, gender, and race. 2) Participants were on hemodialysis or peritoneal dialysis for at least 1 month, and oral MRA (spironolactone or eplerenone) for at least 2 weeks. And patients with a history of kidney transplantation were excluded. 3) One of the following outcomes must have been included: serum potassium (SP), left ventricular mass index (LVMI), left ventricular ejection fraction (LVEF), cardiovascular mortality, all-cause mortality (ACM), systolic blood pressure (SBP) or diastolic blood pressure (DBP). The main characteristics of the included studies are listed in Supplementary Table S1. 4) Only RCTs were included in the meta-analysis.

### Data Extraction

Two reviewers (Li and Jiang) independently extracted data from the same set of publications. The following information were extracted: author, year, sample size, study design, dialysis solution, intervention, control group, follow-up and main results.

### Summary of Effect Size

Odds ratios (OR) with 95% confidence intervals (CIs) were used as the effect size measures of dichotomous data. Mean differences (MD) with 95% CIs were computed for continuous data. The weight of enrolled studies accounted for by taking into account of the size of treatment group, control group, and total sample size. Z-test was calculated and therapeutic efficacy was deemed significant with a *p* < 0.05 cutoff (Li et al., 2019).

### Risk of Bias

The quality of all trials was evaluated independently by two authors (Zhang and Li) according to the Cochrane quality criteria (Fi1 S1). Any disagreement between the authors was settled by discussion with a third author (Tuo).

### Statistical Analysis

STATA 16.0 (Stata Corp LP, College Station, TX, United States) was used to perform statistical analyses. Labbe plot and meta-regression were used for intuitive judgment of heterogeneity. For remaining circumstances, a random effect model was used for pooling the effect size to calculate for statistical heterogeneity. Heterogeneity was analyzed by I^2^ and χ^2^ statistics. If there was significant heterogeneity, a sensitivity analysis was conducted to evaluate the consistency and quality of the results. Publication bias was evaluated using Begg’s and Egger’s tests.

## Results

### Study Selection

A total of 321 literature were identified during the initial search, after excluding duplicate records (n = 35). Thirty-three articles were retained after title/abstract curation (excluding 288 records). Thereafter, we read the full text and enrolled 16 RCTs involving a total of 1,630 patients for quantitative synthesis (Figure 1). The main characteristics of the included RCTs (country, design, sample size, intervention, follow-up and main results) are described in Supplementary Table S1.

### All-Cause Mortality

**FIGURE 2:**
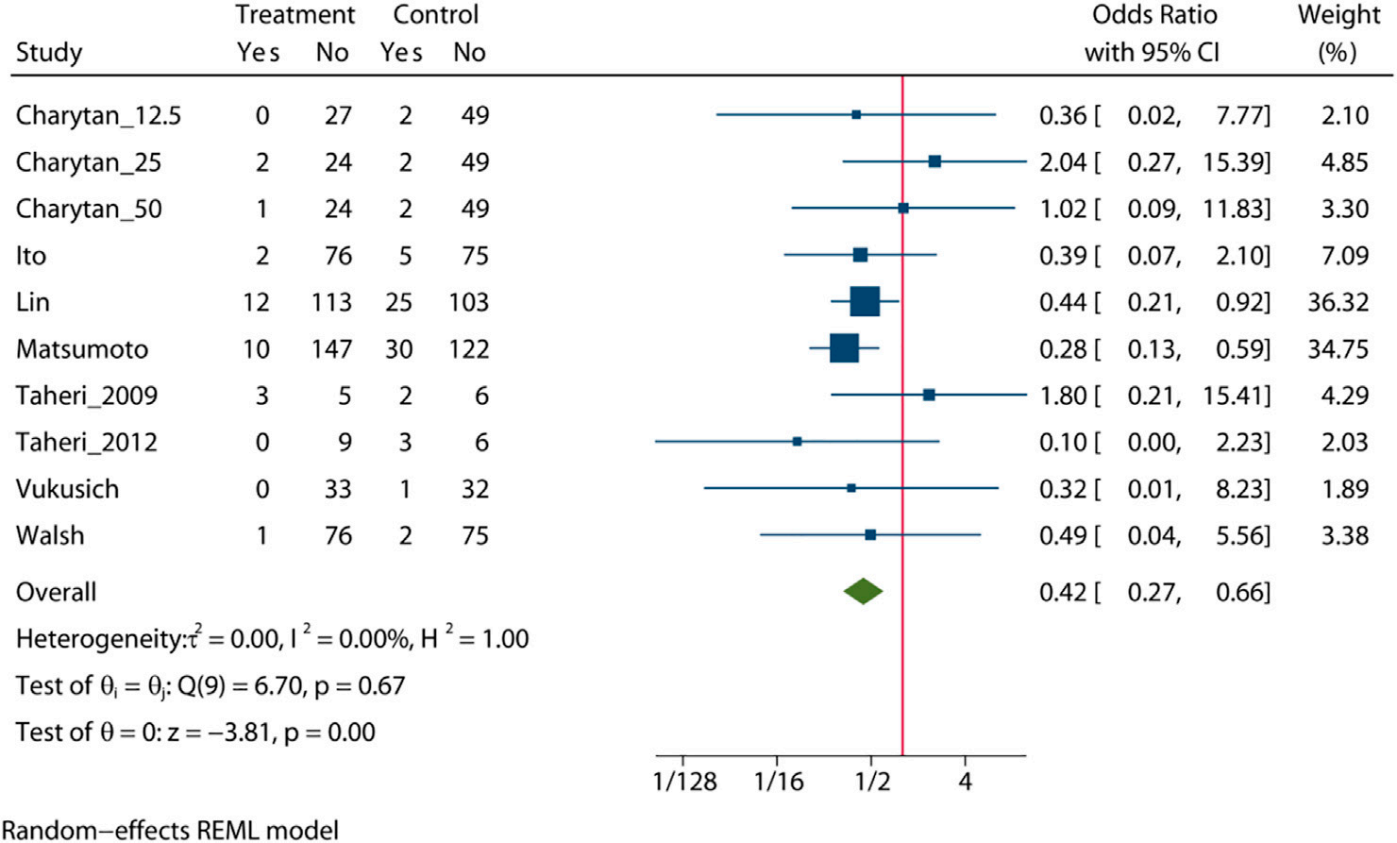
All-cause mortality among patients with MRA vs. control.

**FIGURE 3:**
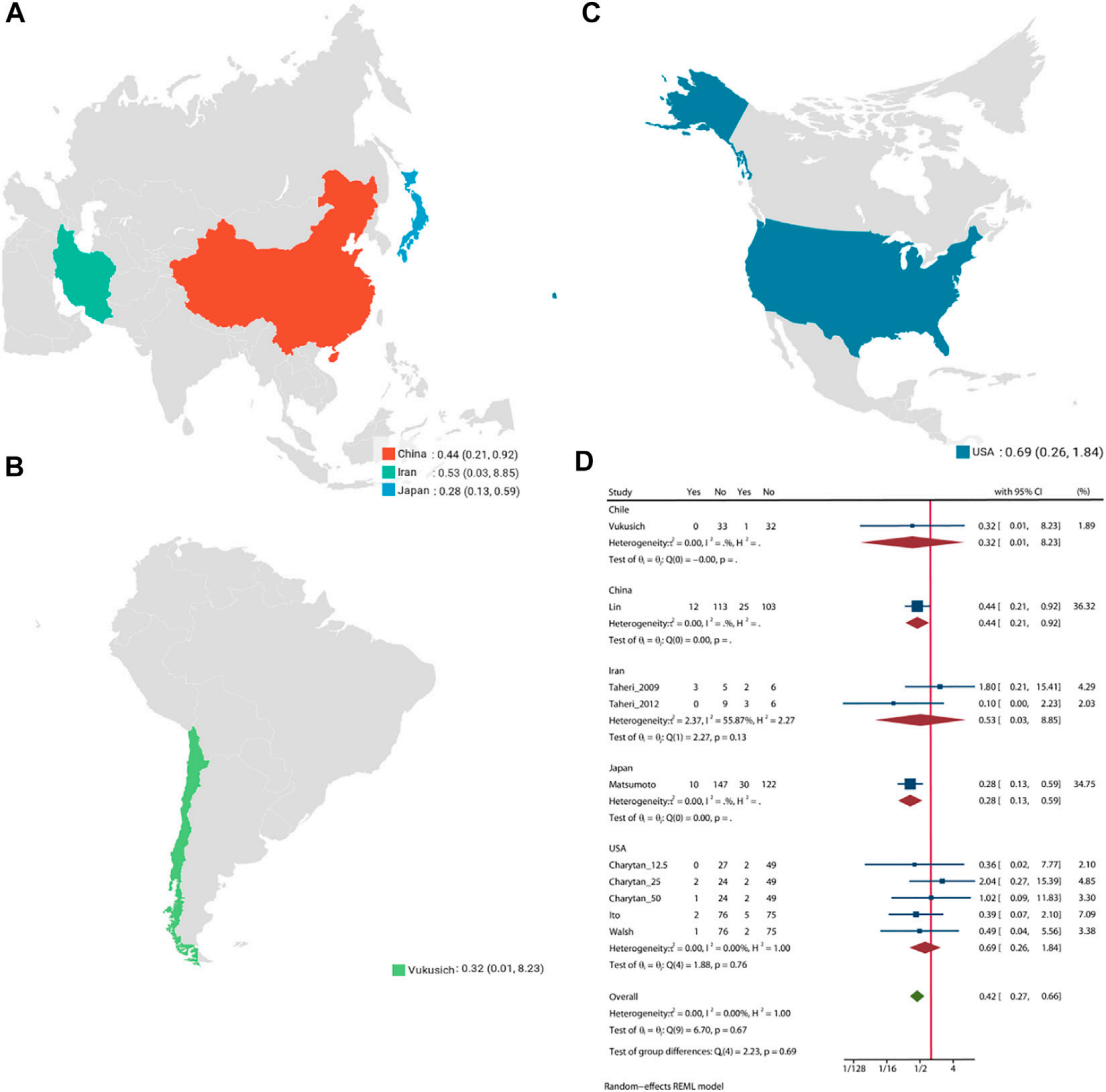
Choropleth map of all-cause mortality.

Pooled analysis of 8 studies (n = 1,205) demonstrates that treatment with MRA (Taheri et al., 2009; Vukusich et al., 2010; Taheri et al., 2012; Ito et al., 2014; Matsumoto et al., 2014; Walsh et al., 2015; Lin et al., 2016; Charytan et al., 2019) may significant reduce the risk of all-cause mortality (OR = 0.42, 95% CI: 0.27, 0.66, *p* < 0.01, Figure 2) versus control. No significant heterogeneity was observed (I^2^ = 0%, Figure 2). The choropleth map reveals that regional difference of all-cause mortality in Japan, Iran, China, Chile and United States (Figure 3). Overall, MRA treatment significantly reduced all-cause mortality versus control in dialysis patients in most Asian countries, but the difference was not significant in Iran, which may be due to the small sample size included in the Iranian study. In North America and South America, the effect of MRA treatment with all-cause mortality on dialysis patients were not significant. Subgroup analysis showed that all-cause mortality was significantly reduced in patients who received both hemodialysis and peritoneal dialysis, but no significant difference was observed in patients who received either hemodialysis or peritoneal dialysis alone (Supplementary Figure S2). Subgroup analysis also found that there was a significant reduction in all-cause mortality in spironolactione group, while there was no significant difference in elperenone group (Supplementary Figure S3).

The meta-regression by Bubble plot reveals no significant heterogeneity of the study size (I^2^ = 0%, *p* = 0.116, Supplementary Figure S4), and publication year was a potential major source of heterogeneity (I2 = 0%, *p* = 0.039, Supplementary Figure S5). Sensitivity analysis was performed to evaluate the stability of our results (Supplementary Figure S6). The analysis results suggested that no individual studies significantly affected the pooled OR, indicating that the results were statistically robust.

### Cardiovascular Mortality

**FIGURE 4:**
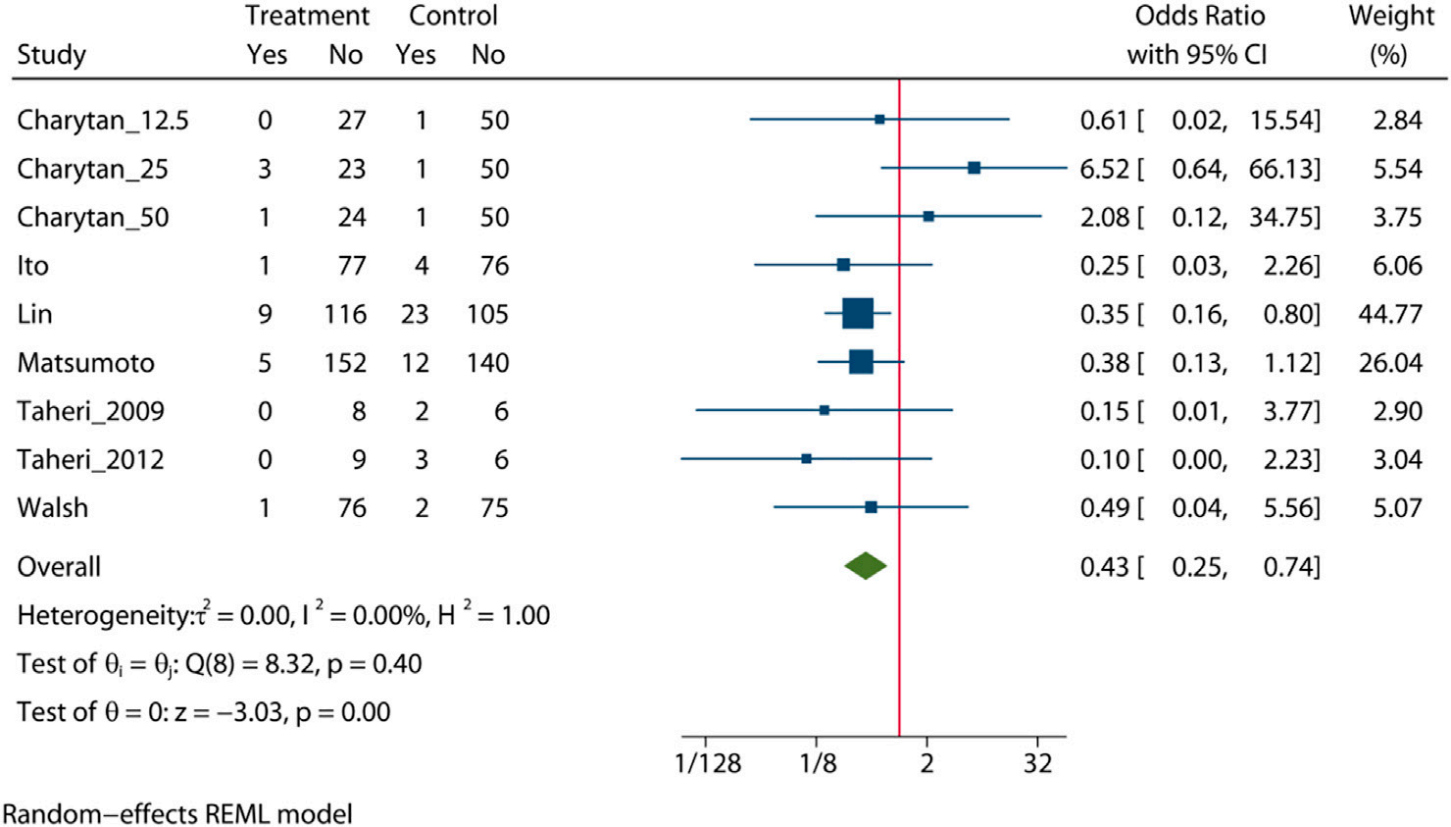
Forest plot of cardiovascular mortality.

**FIGURE 5:**
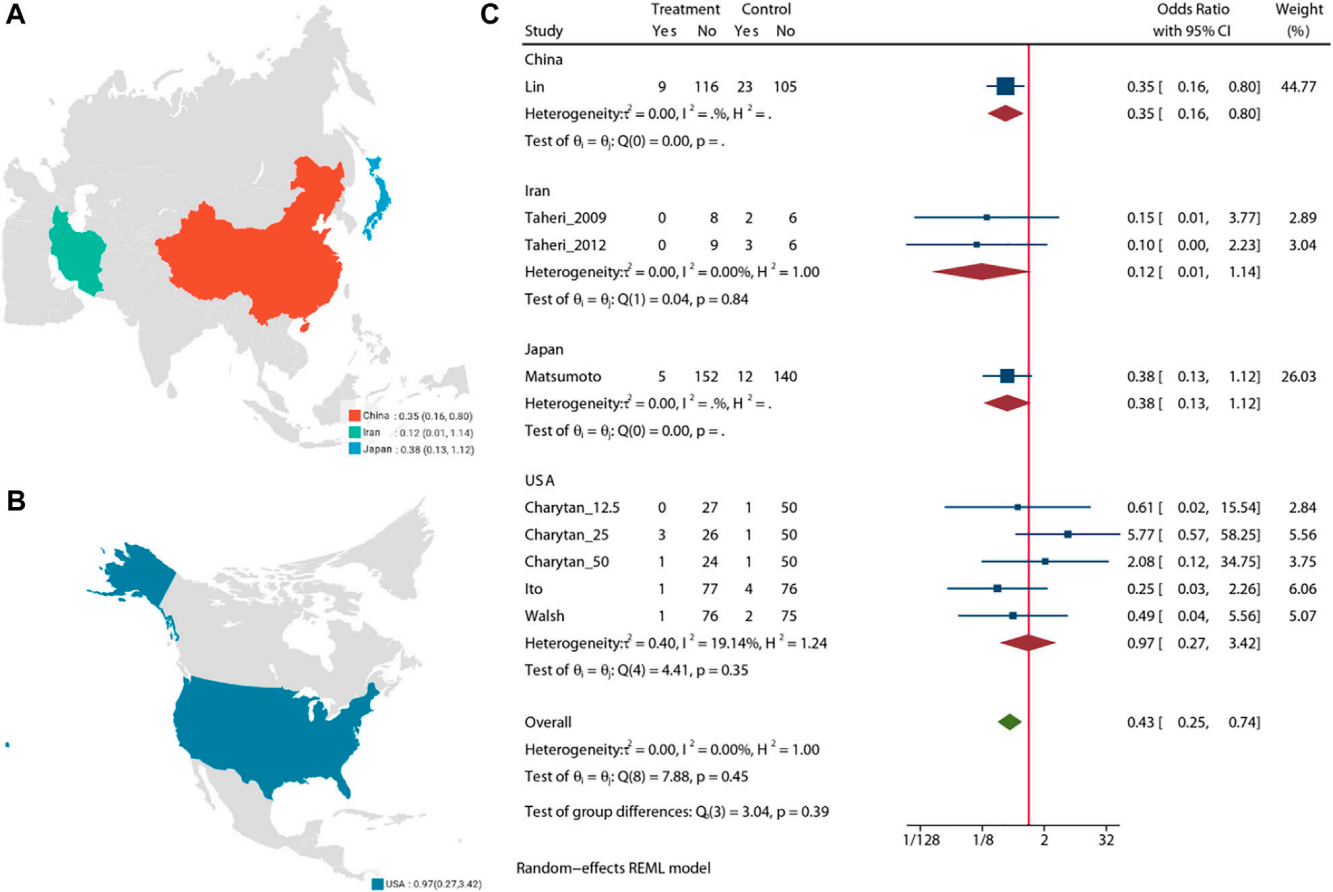
Choropleth map of cardiovascular mortality.

Pooled analysis of 7 studies (n = 988) demonstrates that the MRA treatment (Taheri et al., 2009; Taheri et al., 2012; Ito et al., 2014; Matsumoto et al., 2014; Walsh et al., 2015; Lin et al., 2016; Charytan et al., 2019) may significant reduce the risk of cardiovascular mortality (OR = 0.43, 95% CI: 0.25, 0.74, *p* < 0.01) (Figure 4). No significant heterogeneity was observed (I^2^ = 0%, Figure 4). The choropleth map reveals that regional difference of all-cause mortality in Japan, Iran, China and United States (Figure 5). Overall, MRA treatment significantly reduced the risk of cardiovascular mortality versus control in dialysis patients in China, but the difference was not significant in Japan, Iran and United States. Subgroup analysis showed that cardiovascular mortality was significantly reduced in patients who received both hemodialysis and peritoneal dialysis, but no significant difference was observed in patients who received either hemodialysis or peritoneal dialysis alone (Supplementary Figure S7). Subgroup analysis also found that there was a significant reduction in cardiovascular mortality in spironolactione group, while there was no significant difference in elperenone group (Supplementary Figure S8).

The meta-regression by Bubble plot reveals no significant heterogeneity of the study size (I^2^ = 0%, *p* = 0.134, Supplementary Figure S9), and publication year was a potential major source of heterogeneity (I2 = 0%, *p* = 0.446, Supplementary Figure S10). Sensitivity analysis was performed to evaluate the stability of our results (Supplementary Figure S11). The analysis results suggested that no individual studies significantly affected the pooled OR, indicating that the results were statistically robust.

### Left Ventricular Ejection Fraction

Three RCTs (Feniman-De-Stefano et al., 2015; Charytan et al., 2019; Hammer et al., 2019) involving 345 patient shown that MRA lead to a significant increase in LVEF (MD = −2.71%, 95% CI (−4.86 to −0.56), *p* = 0.01). No significant heterogeneity was observed (I^2^ = 47.9%, Supplementary Figure S12, Supplementary Table S2).

### Left Ventricular Mass Index

Compared with the control groups, MRA treatment (Feniman-De-Stefano et al., 2015; Wang et al., 2018; Charytan et al., 2019; Hammer et al., 2019) significantly reduced the left ventricular mass index (LVMI) (MD = 8.58, 95% CI: 3.69, 13.46, *p* < 0.01). No significant heterogeneity was observed (I^2^ = 40.8%, Supplementary Figure S13, Supplementary Table S2).

### Blood Pressure

The results indicated that additional MRA significantly decreased the systolic blood pressure (SBP) (Feniman-De-Stefano et al., 2015; Gross et al., 2005; Hammer et al., 2019; Yongsiri et al., 2015; Vukusich et al., 2010; Jie et al., 2019; Ni et al., 2014; Tang, 2019) (MD = −7.40 mmhg, 95% CI: 10.60 to −4.20, *p* < 0.01, I^2^ = 27.2%, Supplementary Figure S14, Supplementary Table S2) and diastolic blood pressure (DBP) (Gross et al., 2005; Vukusich et al., 2010; Ni et al., 2014; Feniman-De-Stefano et al., 2015; Yongsiri et al., 2015; Hammer et al., 2019; Jie et al., 2019; Tang, 2019) (MD = 4.60 mmhg, 95% CI: 9.10, 0.10, *p* = 0.04, I^2^ = 81.7%, Supplementary Figure S15, Supplementary Table S2). As there was substantial heterogeneity in DBP, a sensitivity analysis was performed to assess the influence of each individual study (Supplementary Figure S16). The results suggested that no individual study significantly affected the pooled OR, indicating that the results were statistically robust. No significant publication bias was found according to Begg’s plot (*p* = 0.386, Supplementary Figure S17) and Egger’s test (*p* = 0.590, Supplementary Figure S18).

### Serum Potassium Levels

**FIGURE 6:**
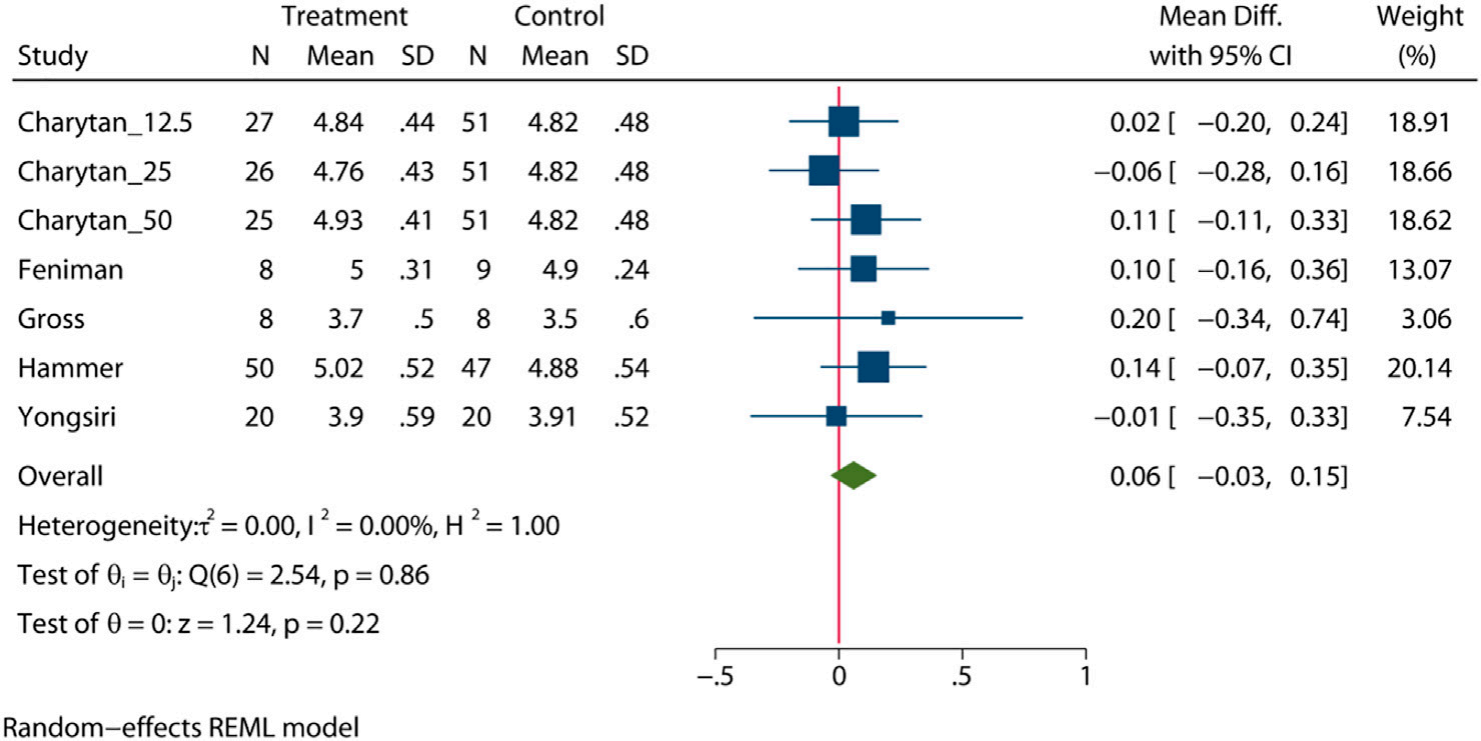
Forest plot of serum potassium level.

To comprehensively assess the influence of MRA, five RCTs (Gross et al., 2005; Feniman-De-Stefano et al., 2015; Yongsiri et al., 2015; Charytan et al., 2019; Hammer et al., 2019) (n = 401) were enrolled to provide an overall estimate of the serum potassium levels after intervention. The statistical analysis showed that there was no significant difference in the serum potassium level between the MRA group and the placebo group (MD = 0.06mEq/l, 95% CI: 0.03 to 0.15, *p* = 0.22, I^2^ = 0%, Figure 6). Subgroup analysis showed that there was no significant difference in the serum potassium level in patients who received either hemodialysis or peritoneal dialysis (Supplementary Figure S19, Supplementary Table S2).

## Discussion

### Main Findings

Compared with previous articles, the biggest innovation of our paper is focus on the benefits of MRA on all-cause mortality and cardiac mortality in dialysis patients. In addition, effects on cardiac function (including SBP, DBP, LVMI and LVEF) and serum potassium were assessed. Our meta-analysis found that MRA vs. control exerts definite cardiovascular effects in dialysis patients, including reducing mortality and cardiovascular mortality, LVMI, and arterial blood pressure, and improving LVEF. And MRA for dialysis patients is with safety that did not increase serum potassium levels.

### Interpretation

It was once believed that aldosterone was secreted by adrenal cortical globular zone cells and that its regulation depended on the classic RAAS pathway (Connell and Davies, 2005). However, in recent years, studies have found that in extra-adrenal tissues, such as heart, brain, liver and vascular endothelium, there is also an autonomous RAAS activation system that induces the synthesis of aldosterone and plays an important role in local tissues (Gómez-Sánchez et al., 1996; Silvestre et al., 1998; Slight et al., 1999). Therefore, the inhibition of RAAS and the reduction of aldosterone levels have been important targets in past clinical interventions for heart reconstruction, HFrEF and hypertension. However, with the widespread application of ACEIs/ARBs in clinical practice, it has been found that even in patients treated with these drugs, the level of aldosterone in blood is still difficult to maintain at a low level, moreover, once the level of aldosterone is no longer decreasing, it gradually increases to pretreatment levels or higher, which indicates the existence of the “aldosterone escape phenomenon” (Struthers, 1996; Lakkis et al., 2003). Therefore, the production of aldosterone and its secondary target, i.e., organ damage, could not be reduced by RAAS blockers alone.

In 2016, Quach (Quach et al., 2016) etc. found that MRA as a potentially beneficial treatment to reduce cardiovascular mortality for dialysis patients, and there is a substantial risk for hyperkalemia that may limit widespread use of MRA in dialysis patients. However, due to a lack of high-quality and large-sample data, their conclusions are debatable. As several recent RCTs have been published, the current meta-analysis aimed to re-evaluate the benefits and risks of MRA in patients undergoing hemodialysis. In 2021, Hasegawa (Hasegawa et al., 2021) etc. found that MRA probably reduces the risk of all-cause and cardiovascular death and probably reduces morbidity due to cardiovascular and cerebrovascular disease in people with CKD requiring dialysis. This is similar to the conclusion of our paper. Pitt (Pitt et al., 2021) etc. found that finerenone is a novel, selective, nonsteroidal MRA that blocks MR-mediated sodium reabsorption and MR overactivation and has demonstrated anti-inflammatory and anti-fibrotic effects in preclinical kidney and cardiovascular disease models. Finerenone have shown interesting data in CKD in diabetes, but not tested in dialysis yet.

MRAs are one of the cornerstones of therapy in HFrEF (Pitt et al., 1999). However, practical implementation of high-grade trial evidence is hindered by physicians’ apprehension regarding the hyperkalemia risk, which is inherently associated with RAAS blockade. The Spin-D (Safety and Cardiovascular Efficacy of Spironolactone in Dialysis-Dependent End-Stage Renal Disease) and MiREnDa (Mineralocorticoid Receptor Antagonists in End-Stage Renal Disease) trials taken together provide the reassuring demonstration that up to 25 mg/d spironolactone is reasonably safe, provided maintenance hemodialysis patients are properly monitored and investigators use a per-protocol therapeutic algorithm to manage hyperkalemia. These results should encourage and reassure the investigators of the 2 currently ongoing, large, international, major-outcome clinical trials, both of which are using spironolactone up to 25 mg/d: ACHIEVE (Aldosterone bloCkade for Health Improvement EValuation in End-stage Renal Disease trial; NCT03020303) and ALCHEMIST (ALdosterone antagonist Chronic HEModialysis Interventional Survival Trial; NCT01848639) (Rossignol et al., 2019).

Our analysis included a total of 1,630 patients across 16 RCTs. In our study, the results showed that a low dose of spironolactone (12.5–50 mg daily) could significantly improve the LVMI and LVEF of hemodialysis patients, reduce arterial blood pressure and improve the long-term survival rate without significantly increasing the serum potassium level. However, we also noticed that hyperkalemia occurs more frequently as the dosage increases to 50 mg daily. It seems that 25 mg daily is likely to be the best choice to strike a balance between the effects of the drug and its side effects, consistent with the findings of Zannad et al. (Zannad et al., 2000). In addition, recent findings (Noori et al., 2010) have indicated that serum potassium levels are positively correlated with potassium intake in dialysis patients. Therefore, it is also important to strengthen diet education in patients undergoing dialysis to avoid the occurrence of high serum potassium levels.

Hypertension is an important risk factor for cardiovascular disease and is very common in ESRD patients. In a cohort study with a large sample size, the prevalence of hypertension was found to be higher than 80% among patients with ESRD, and only approximately 30% of these patients had good blood pressure control (Agarwal et al., 2003). Currently, it is believed (Xu et al., 2013) that the causes of hypertension in dialysis patients include capacity overload, over activation of the RAAS system, over excitation of the sympathetic nervous system, vascular endothelial injury and so on. In our analysis, it was suggested that the use of MRA during treatment could reduce SBP and DBP in hemodialysis patients, and the antihypertensive effects of MRA were confirmed. This non-diuretic antihypertensive mechanism included reducing sympathetic tension (Rahmouni et al., 2002), regulating endothelial cell function (Sui and Wang, 2002), improving vascular compliance (Sarnak et al., 2003a) and reducing arterial stiffness (Calhoun et al., 2008).

Previous studies have shown that approximately 75% of maintenance hemodialysis patients have left ventricular hypertrophy and 40% have left ventricular insufficiency, and the incidence of cardiovascular and cerebrovascular events is significantly higher than that of normal left ventricular hemodialysis patients (Sarnak et al., 2003b). The recovery of left ventricular structure and function can also improve the prognosis of patients (Tian et al., 2009). Zoccali et al. (Zoccali et al., 2001) found that abnormally high concentrations of blood aldosterone can induce myocardial fibrosis and aggravate ventricular remodeling, while spironolactone can reverse ventricular hypertrophy, improve cardiac function and alleviate HFrEF by antagonizing the fibrosis-promoting effect of aldosterone.

We used the LVMI and LVEF as evaluation criteria for left ventricular hypertrophy and left ventricular systolic function, respectively. Our meta-analysis found that spironolactone can reverse left ventricular hypertrophy and improve LVEF in patients with hemodialysis. In addition to the protective cardiovascular effects of lowering blood pressure and preventing heart remodeling, spironolactone also has unexpected benefits. Currently, it is generally believed that an increase in CIMT is an early manifestation of atherosclerosis, the degree of atherosclerosis can be evaluated early by detecting CIMT with ultrasound, and the risk of adverse cardiovascular and cerebrovascular events can be predicted. Vukusich et al. (Vukusich et al., 2010) found that 50 mg of spironolactone thrice weekly significantly reduced the progression of CIMT in hemodialysis patients. The results demonstrate that spironolactone may have an additional role in reversing atherosclerosis, which may also be related to its anti-vascular remodeling.

We noticed that previous published meta-analyses omitted important trials (e.g., there are only five RCTs in Zhao’s work (Zhao et al., 2016)), Zhao’s conclusion that MRA did not increase potassium levels significantly, this is consistent with our result. However, they did not analyze the effect of MRA on serum potassium in patients with different dialysis methods, we did a subgroup analysis specifically for this question. Zhao’s study found that the effects of MRA on BP, LVMI and LVEF with dialysis patients were controversial, but quantitative analysis was not performed due to insufficient data. In our paper, BP (DBP and SBP included), ACM, cardiovascular mortality, LVMI and LVEF were analyzed respectively, and subgroup analysis were conducted on the effects of different dialysis methods, different MRA drug types and different countries.

Strikingly, the conclusion seems to be opposed to previous publications (Roscioni et al., 2012). Based on previous studies and hypothesis, the following explanations may address this question. 1) The patients type is different. Previous studies may only included patients with single dialysis type, not focus on patients with both hemodialysis and peritoneal dialysis, that would have an impact on their conclusions. 2) Differences in different countries or areas. Since most of the previous studies were single-center studies, the conclusions of different countries may be limited. It can be clearly seen from our subgroup analysis that the conclusions of different countries may be significantly different. 3) Insufficient RCTs were included. Lack of adequate RCTs may lead to publication bias or failure to draw conclusions.

More importantly, our results show that MRA significantly reduce ACM and cardiovascular mortality in hemodialysis patients. As mentioned above, spironolactone has multiple protective cardiovascular effects in patients with hemodialysis, and the possible mechanisms for its beneficial effects on long-term prognosis include diuretic and non-diuretic hypotension, the reversal of left ventricular hypertrophy, the improvement of cardiac systolic function, anti-atherosclerosis and the improvement of heart rate variability in patients undergoing hemodialysis (Michea et al., 2004)^.^

### Strengths and Limitations

Firstly, our meta-analysis was performed by a Cochrane Member and supervised by strict quality control evaluated by Cohen’s kappa coefficient (κ = 0.823, 95% CI: 0.642–0.937), which showed a high degree of agreement. Secondly, our study included 16 RCTs with more than 1,630 participants and conducted a comprehensive and thorough assessment of the risks of publication bias, sensitivity analyses, subgroup analyses, Begg’s plot, Egger’s test and meta-regression to ensure that the results were reliable. we attempted to be as inclusive and transparent in this manuscript in terms of our methods including all origin of software and websites. Thirdly, we refined our analyses strictly in accordance to the PICOS principle. Specifically, we conducted 5 subgroup analysis perform the effects of different dialysis methods, different MRA drug types and different countries, and refined the outcomes into 10 types.

However, several limitations of our meta-analysis shall be considered. First, the true event rates of participants lost to follow-up are unpredictable and unlikely to be at either extreme of our assumptions. Second, the quality of several trials was not high, follow-up times were different, and the treatment doses were variable, which could have affected our conclusions. Third, the concomitant diseases of patients included in various institutes were different, which may lead to a risk of bias. Fourth, this meta-analysis did not include a particularly large number of RCTs and could have affected our conclusions. Despite statistical significance, the results should be interpreted cautiously, and further studies are needed to substantiate or refute these results before any changes to clinical practice or guidelines are made.

## Conclusion and Implications

Our meta-analysis demonstrated that MRA can effectively improve the dialysis patient’s heart function, reduce incidence of cardiovascular morbidity and mortality, including reducing mortality and cardio mortality, LVMI, and arterial blood pressure, and improving LVEF. and does not significant affect the potassium level. We hope that the results of our study will provide some reference for the prevention and treatment of cardiovascular diseases in dialysis patients.

## Data Availability

The original contributions presented in the study are included in the article/Supplementary Material, further inquiries can be directed to the corresponding authors.
