# Supplementary material for: Association of Mineralocorticoid Receptor Antagonists With the Mortality and Cardiovascular Effects in Dialysis Patients: A Meta-analysis
Source: Front Pharmacol. 2022 May 17;13:823530. doi: 10.3389/fphar.2022.823530 (PMC9152260; doi:10.3389/fphar.2022.823530)
Supplement: Supplementary file 1 [file DataSheet1.docx]

**Fig S1. Risk of bias of included studies.**


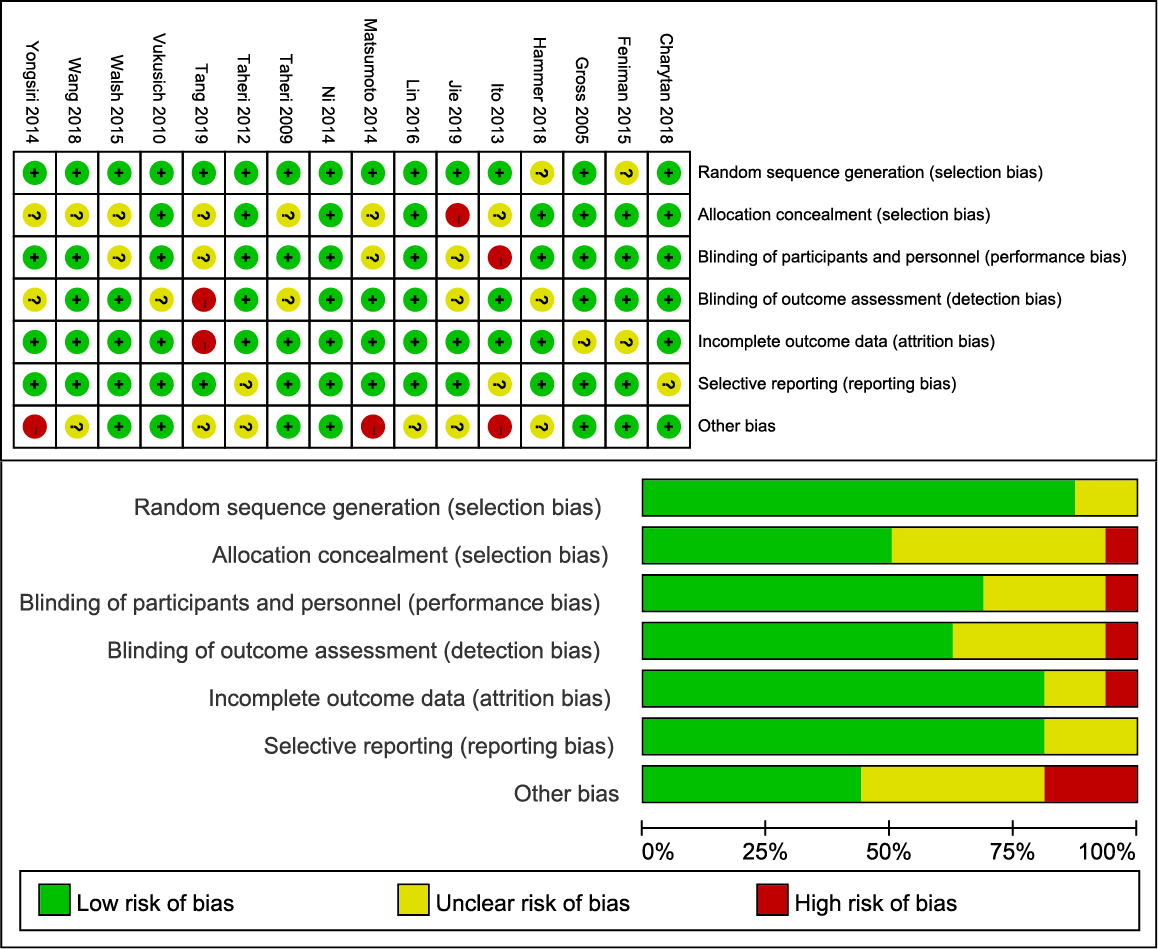


**Fig S2. All-cause mortality among patients with different types of dialysis.**

**
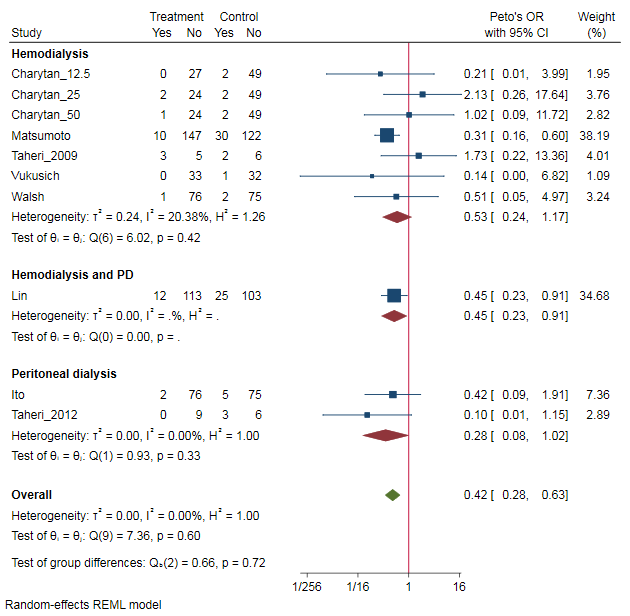
**

**Fig S3. All-cause mortality among patients with spironolactone vs eplerenone treatment.**

**
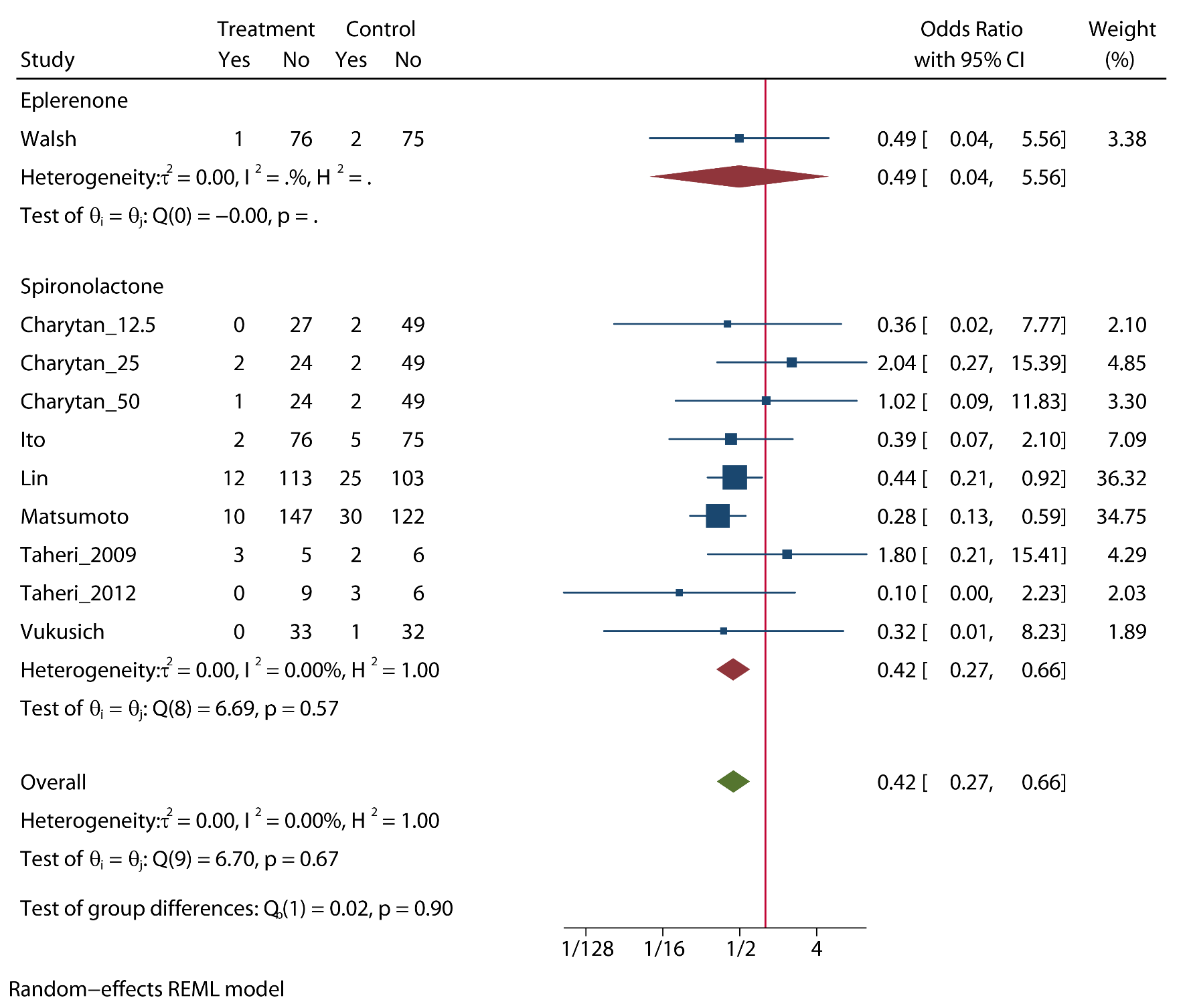
**

**Fig S4. Meta-regression of year of all-cause mortality.**

**
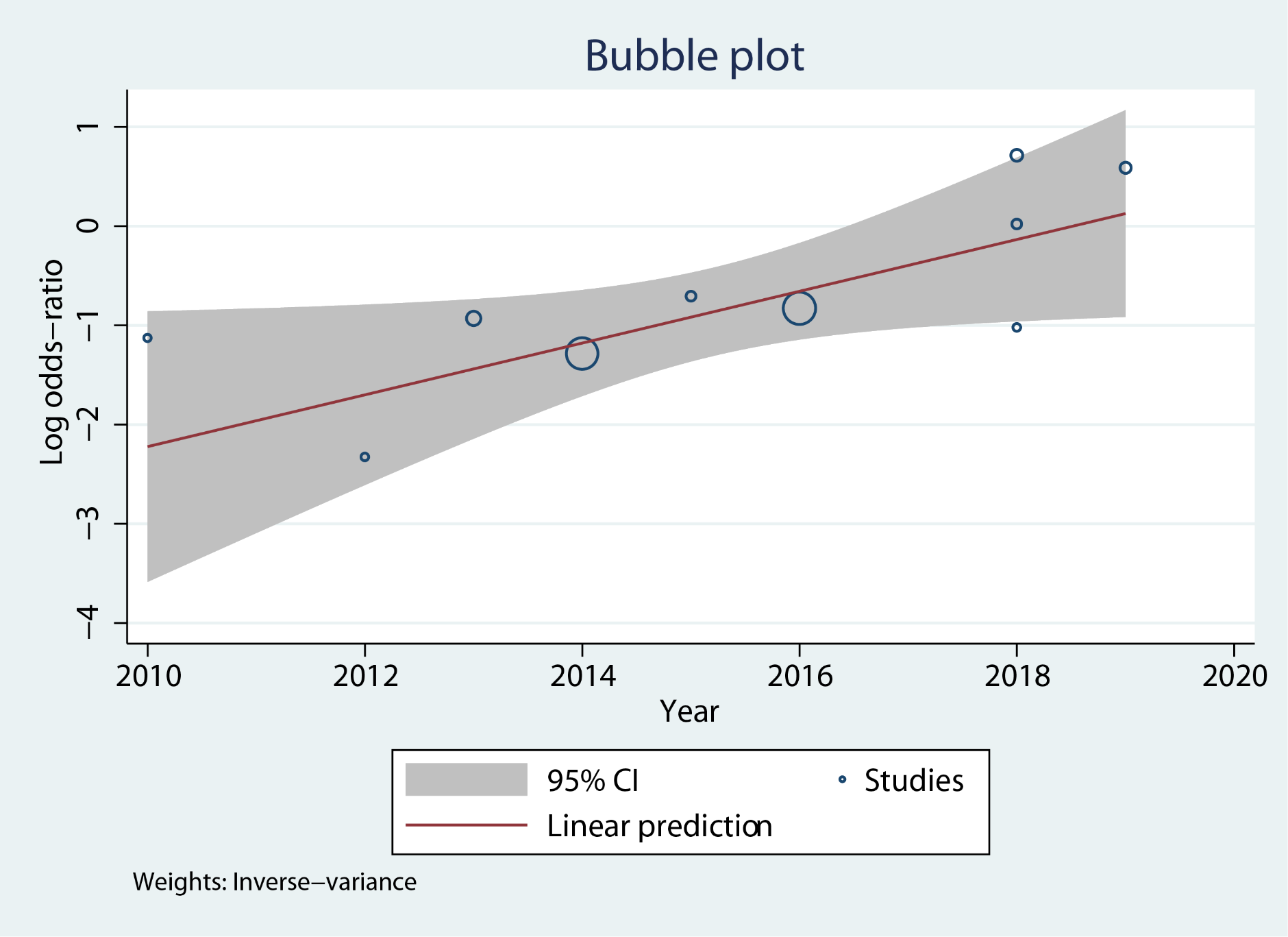
**

**Fig S5. Meta-regression of study size of all-cause mortality.**

**
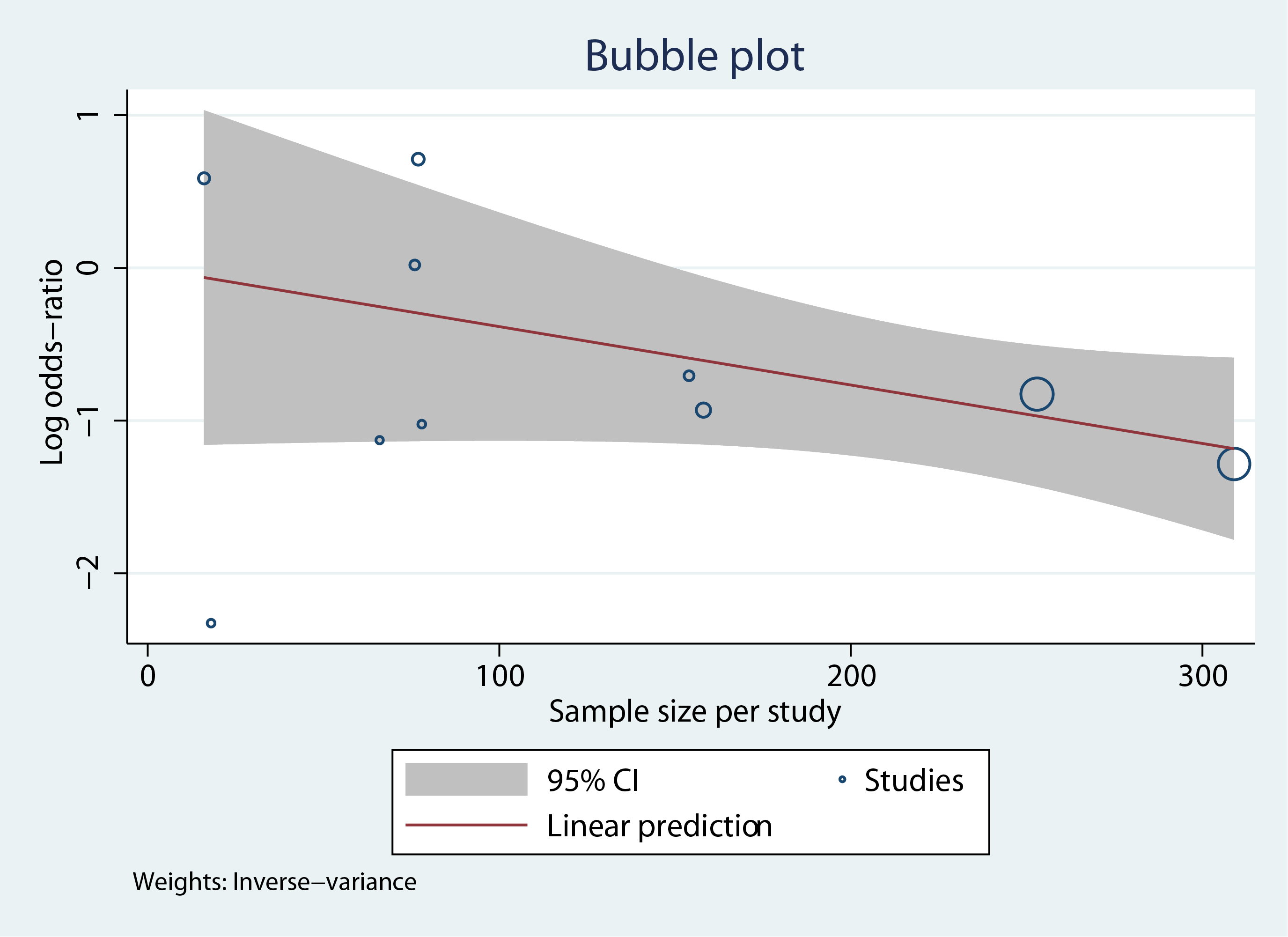
**

**Fig S6. Sensitivity analysis for all-cause mortality.**

**
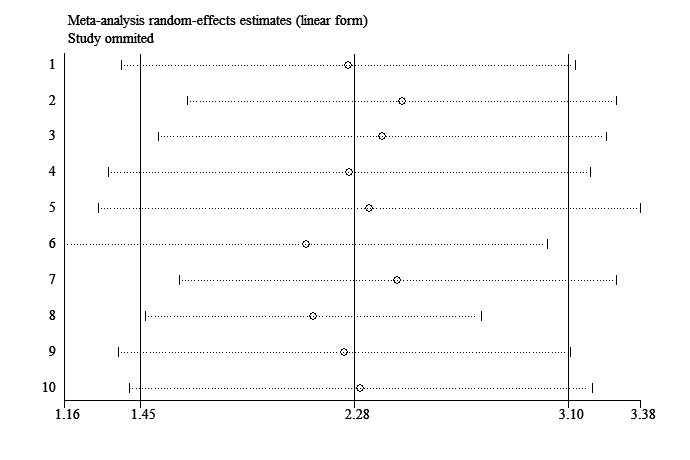
**

**Fig S7. Cardiovascular mortality among patients with hemodialysis vs peritoneal dialysis.**

**
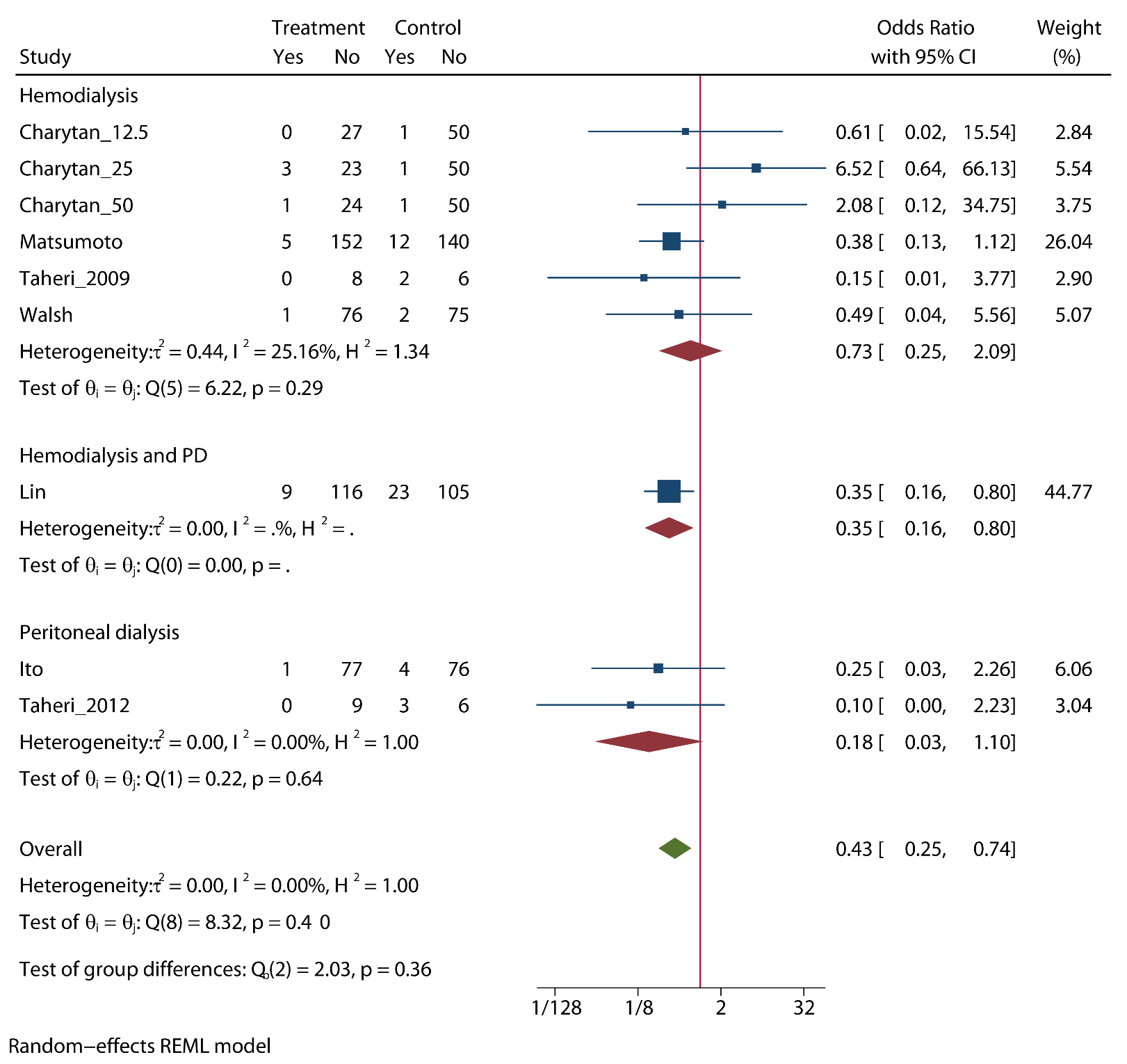
**

**Fig S8. Cardiovascular mortality among patients with oral spironolactone vs eplerenone treatment.**

**
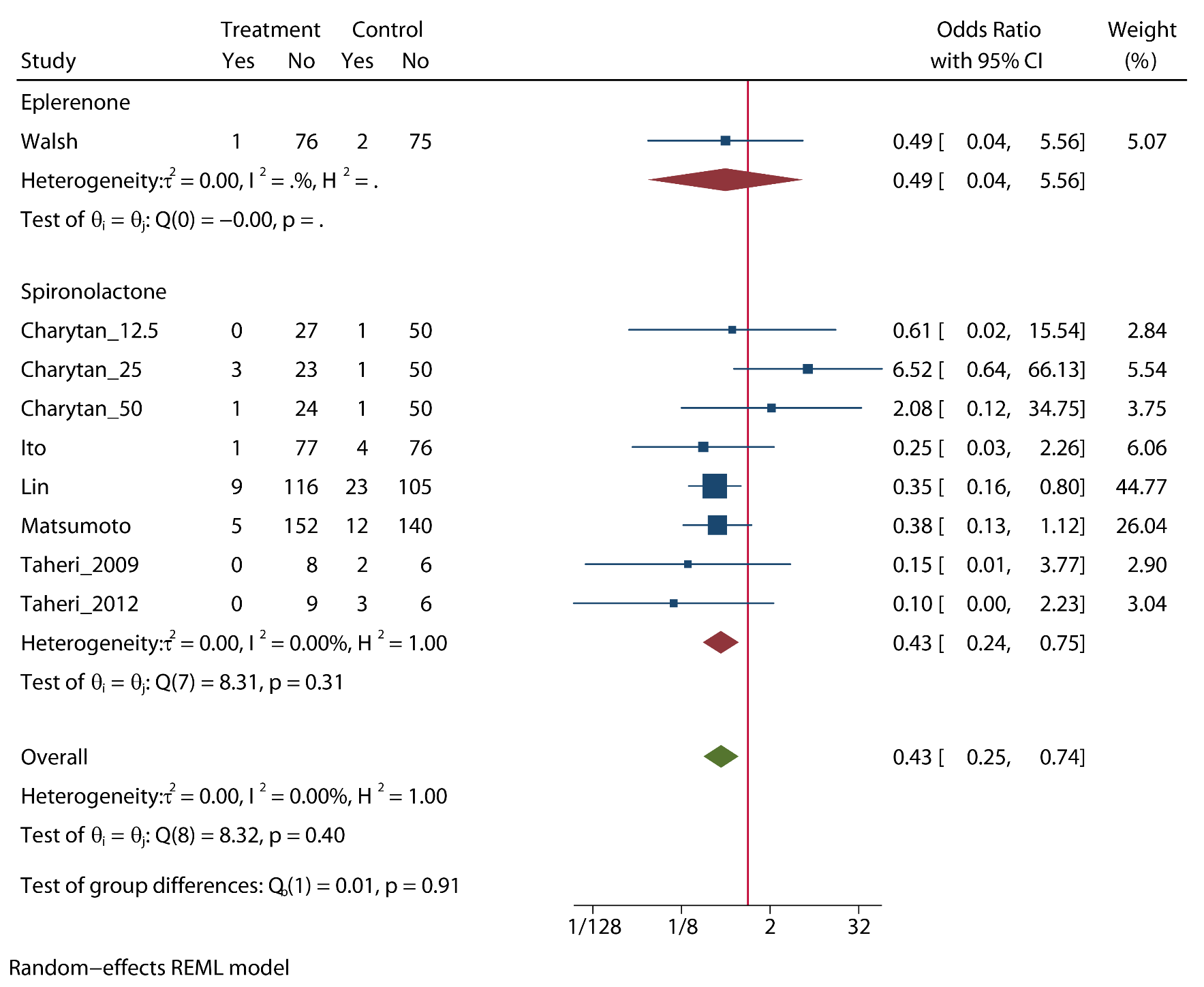
**

**Fig S9. Meta-regression of year of cardiovascular mortality**

**
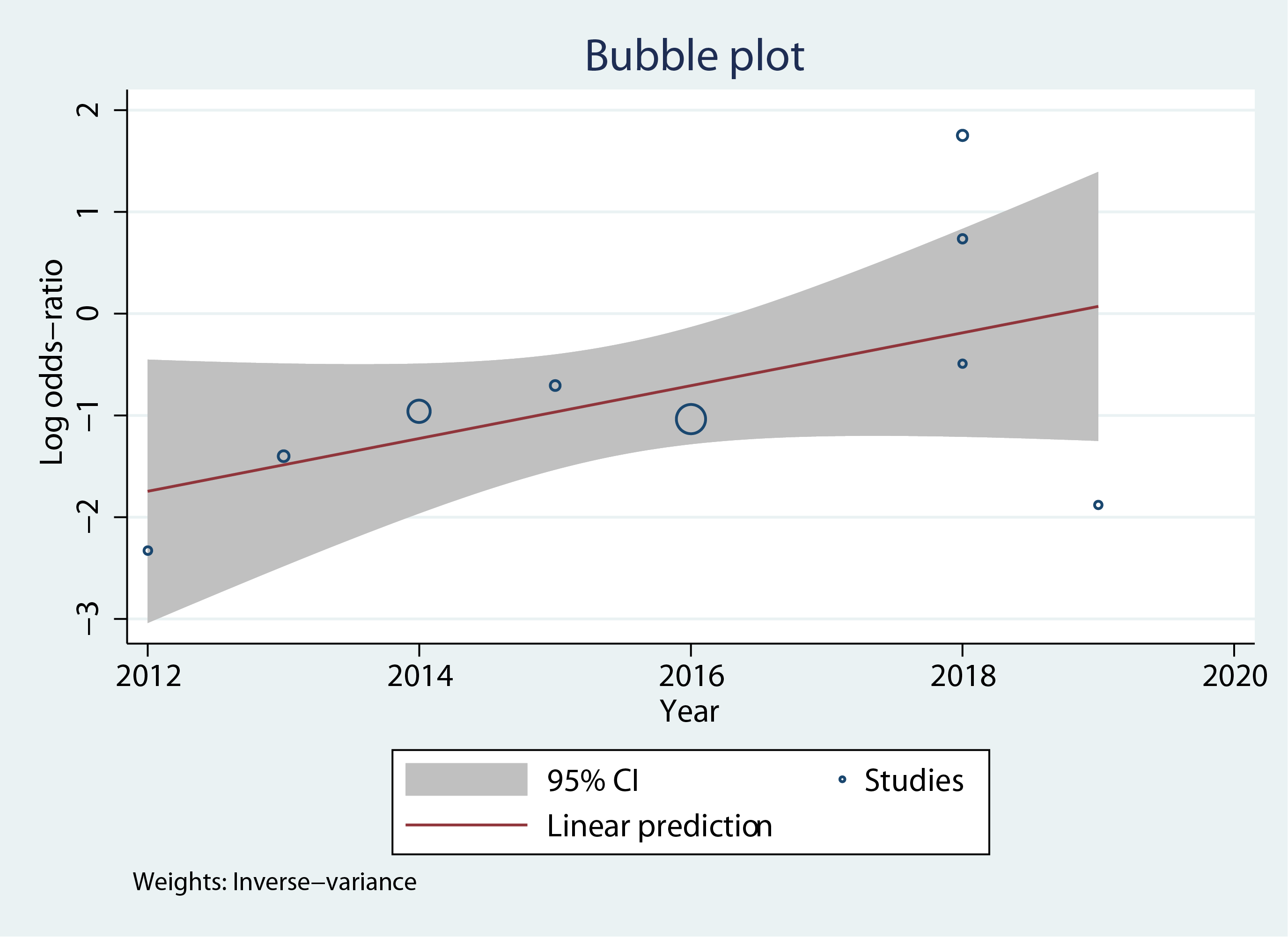
**

**Fig S10. Meta-regression of study size of cardiovascular mortality**

**
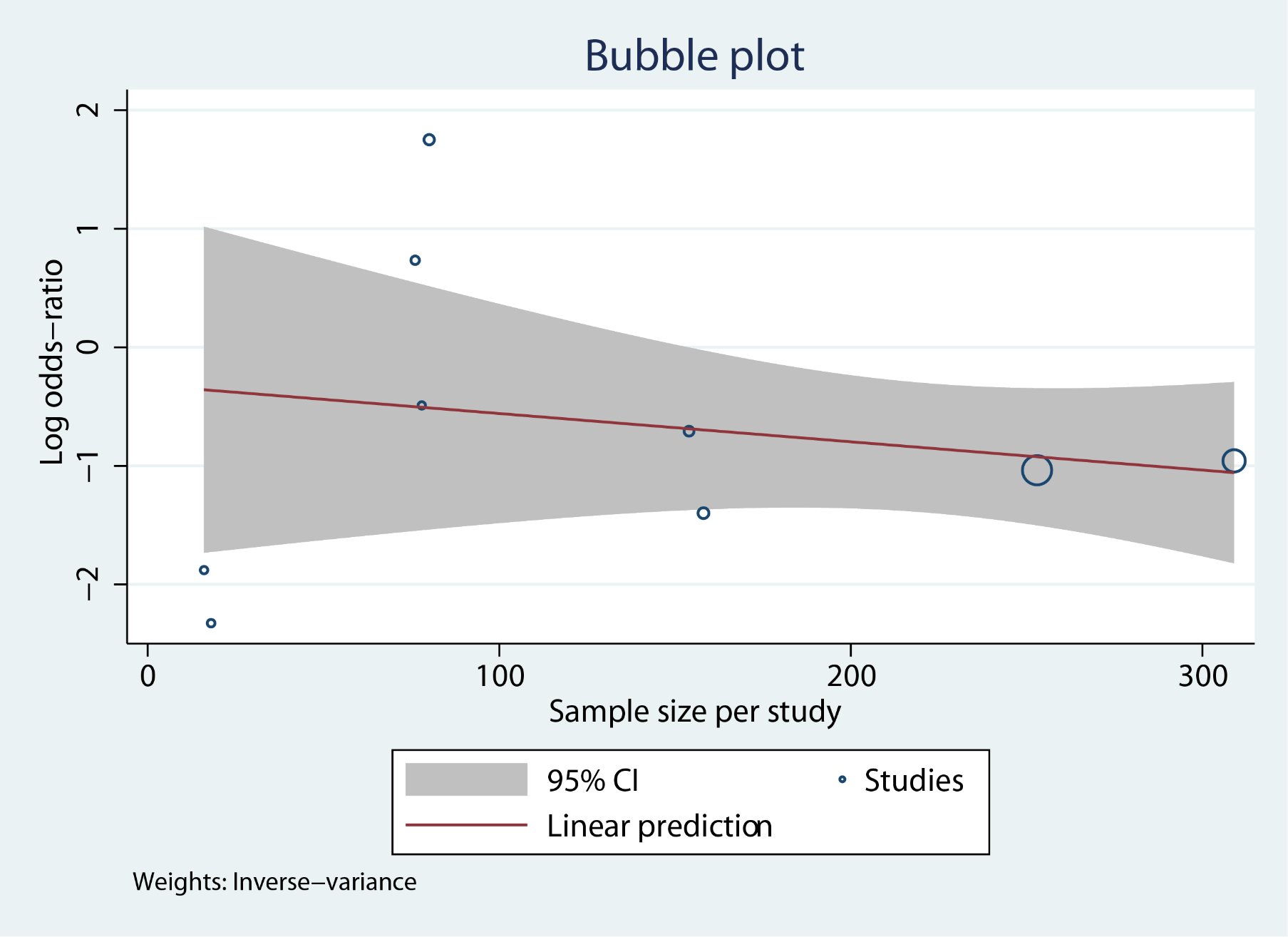
**

**Fig S11. Sensitivity analysis for cardiovascular mortality**

**
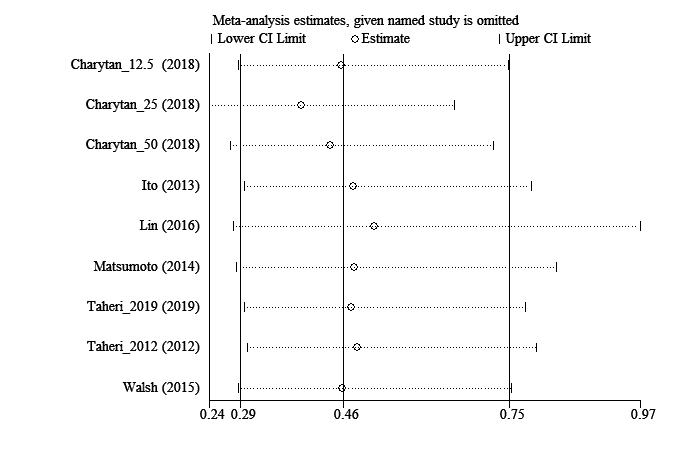
**

**Fig S12. Left ventricular ejection fraction among patients with MRA vs control.**

**
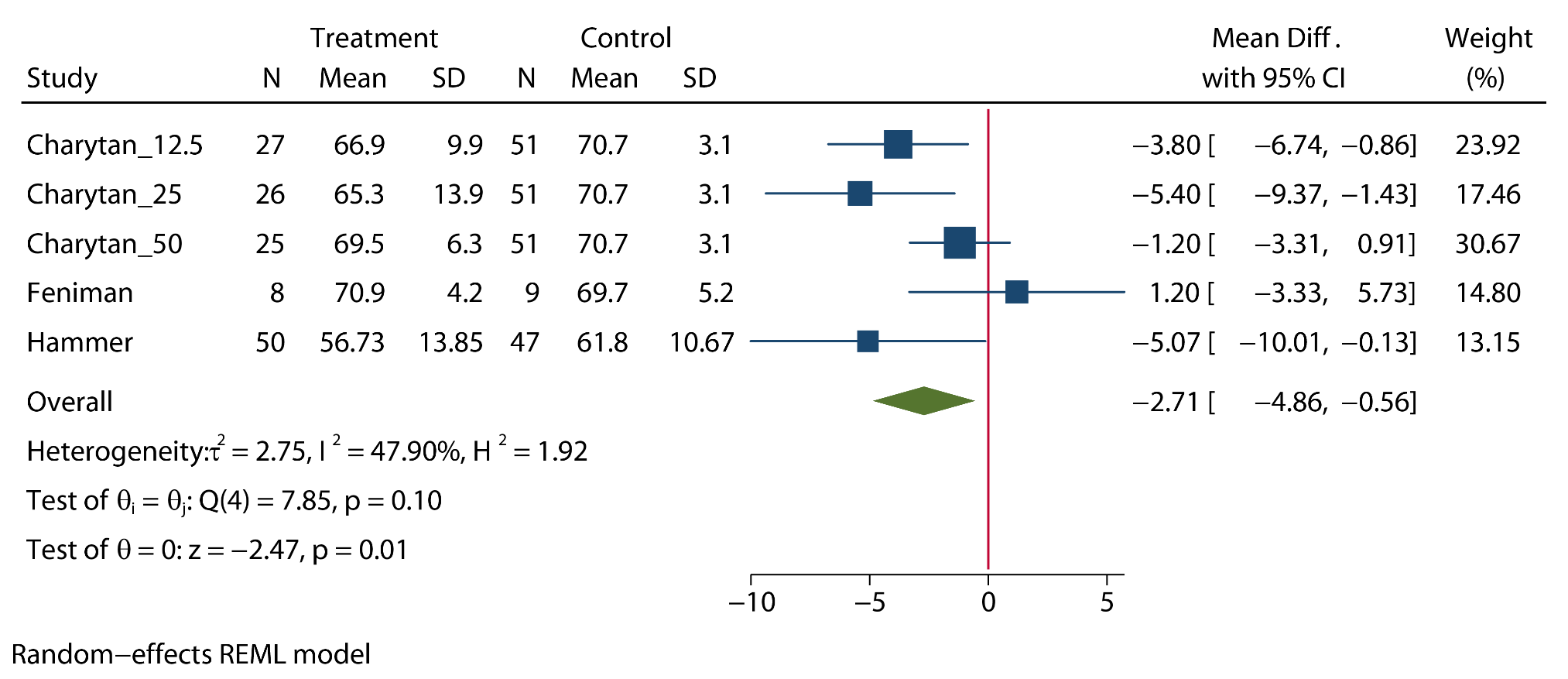
**

**Fig S13. Left ventricular mass index among patients with MRA vs control.**

**
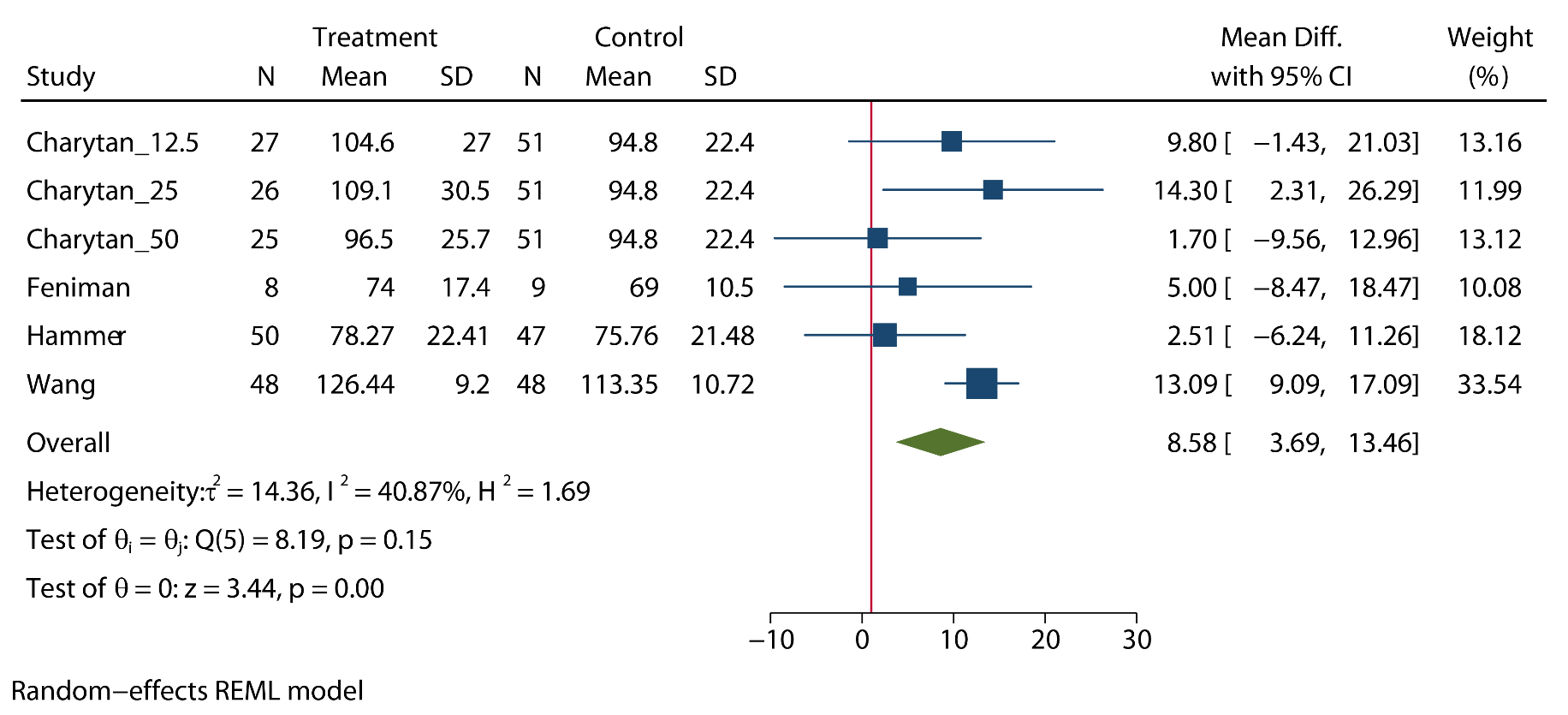
**

**Fig S14. Systolic blood pressure among patients with MRA vs control.**

**
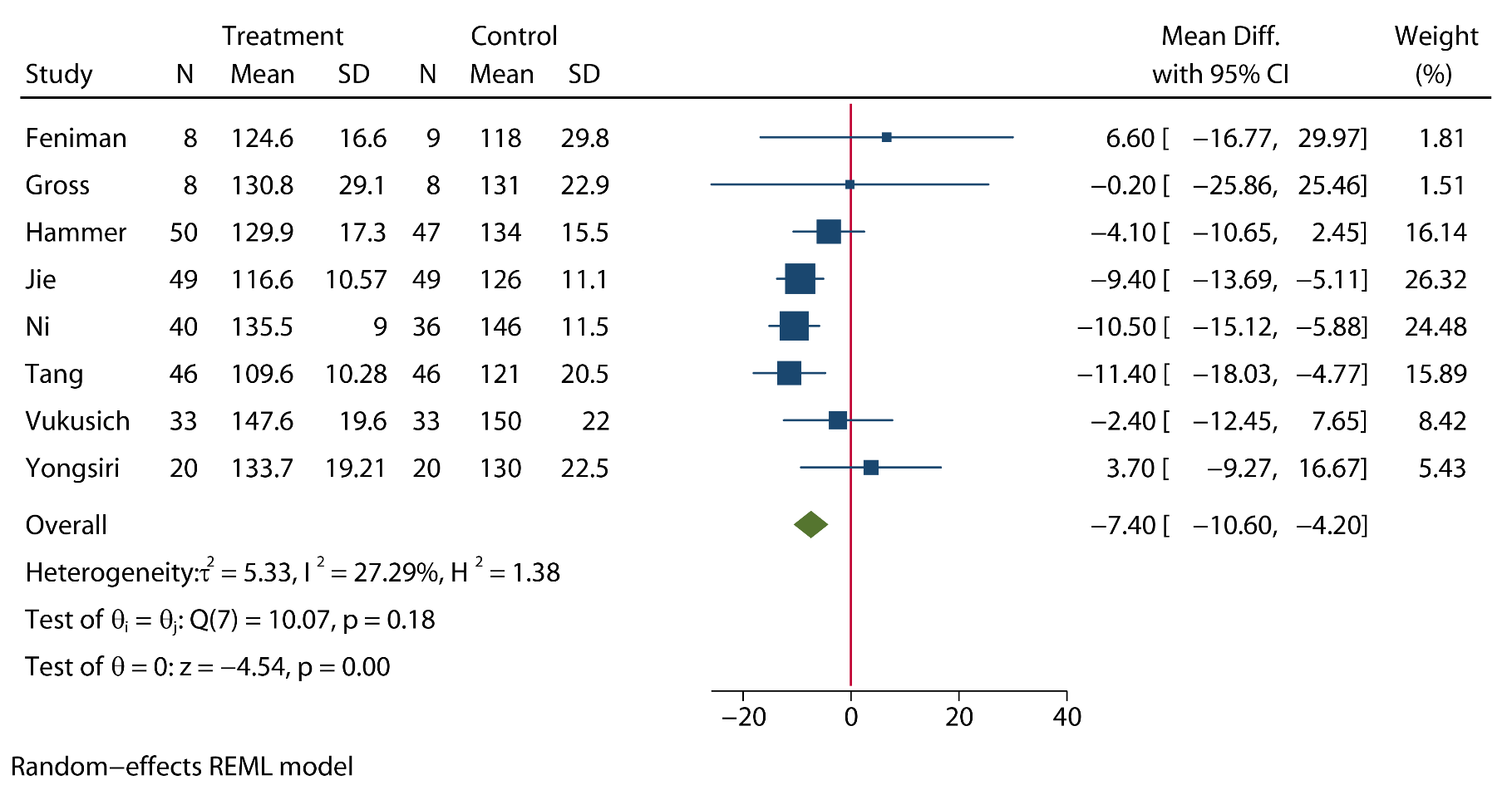
**

**Fig S15. Diastolic blood pressure among patients with MRA vs control.**

**
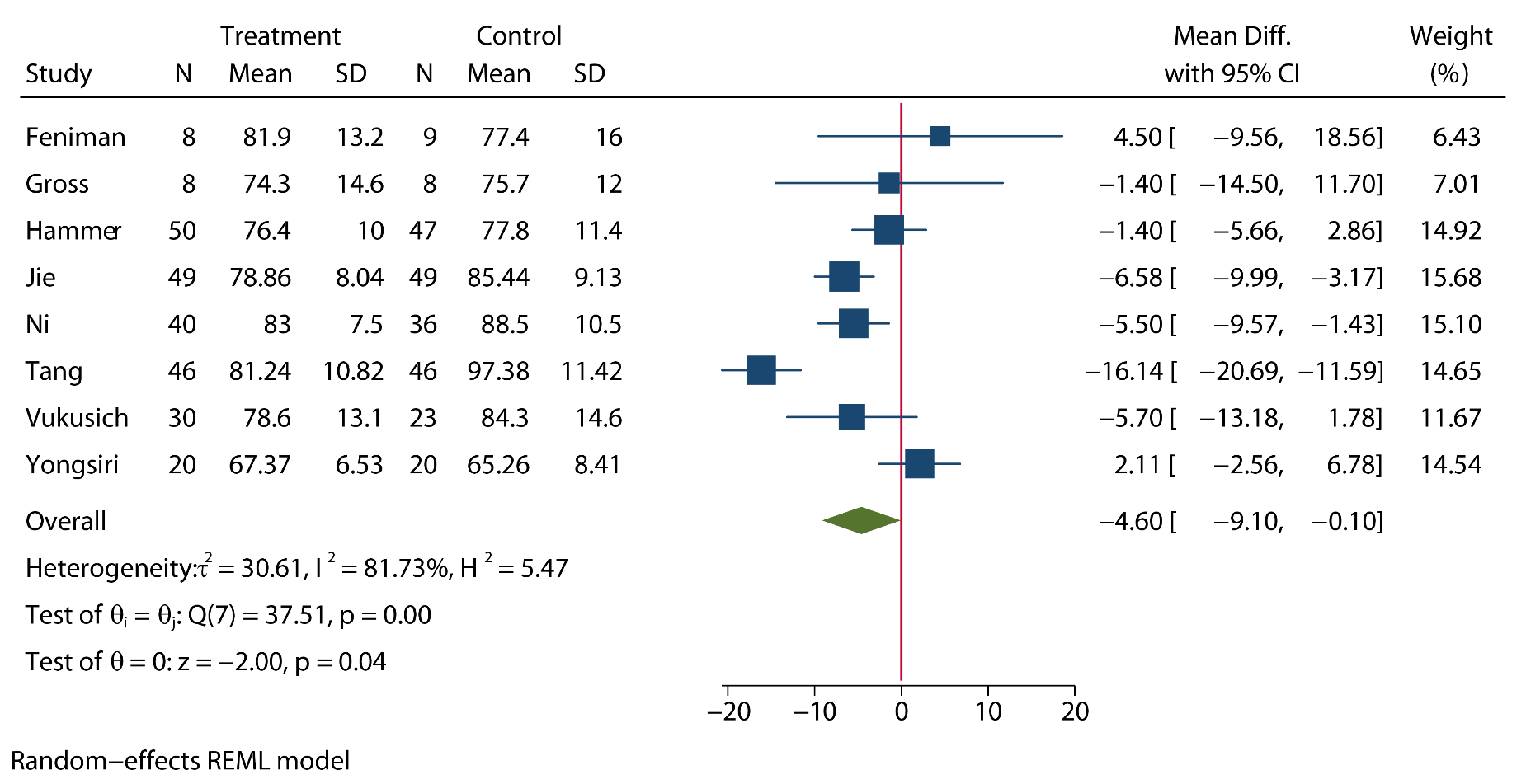
**

**Fig S16. Sensitivity analysis for diastolic blood pressure.**

**
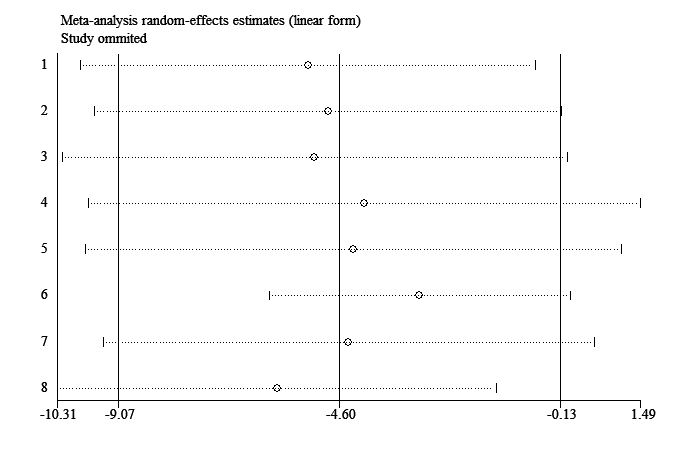
**

**Fig S17. Begg's funnel plot for diastolic blood pressure**

**
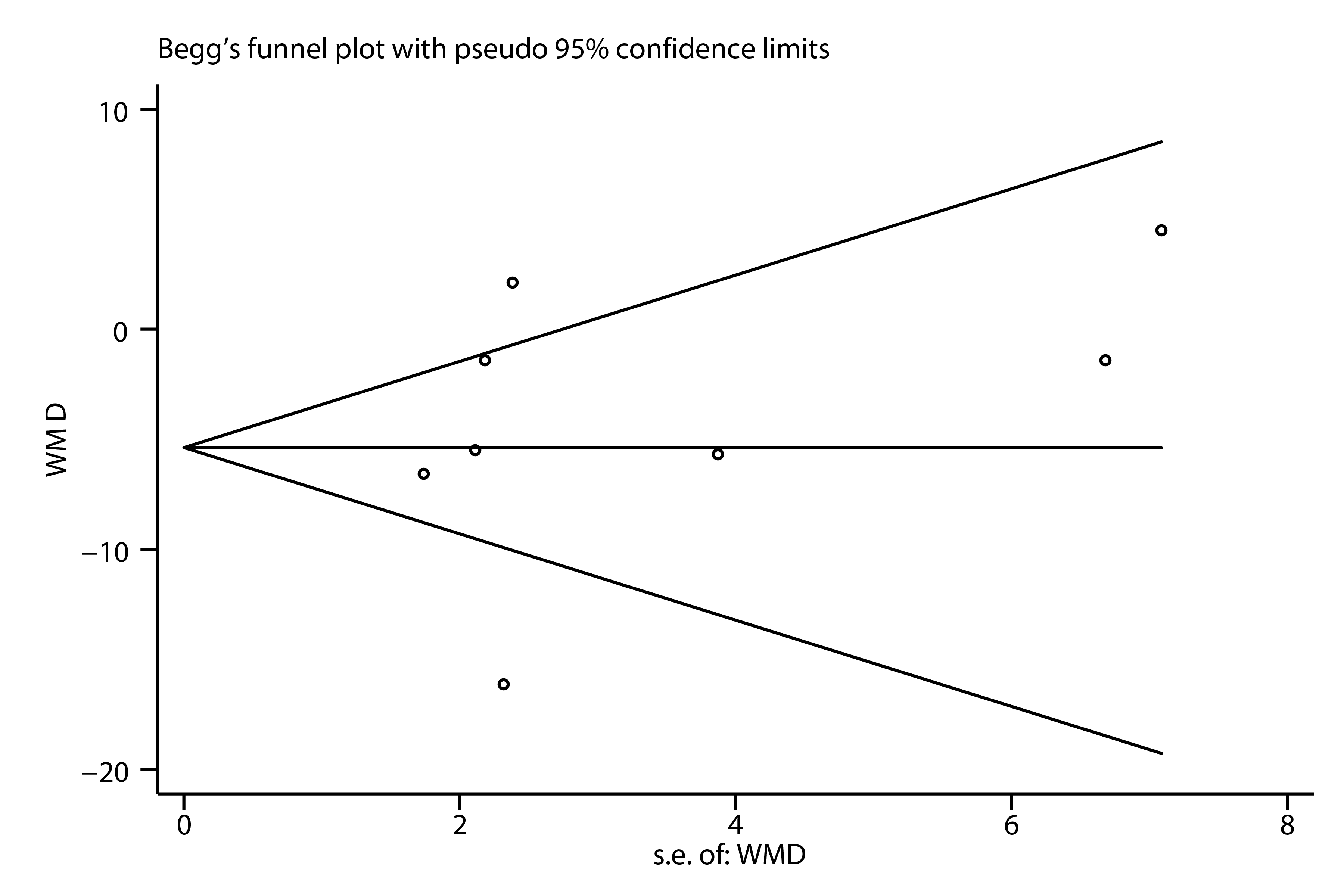
**

**Fig S18. Egger's publication bias plot for diastolic blood pressure**

**
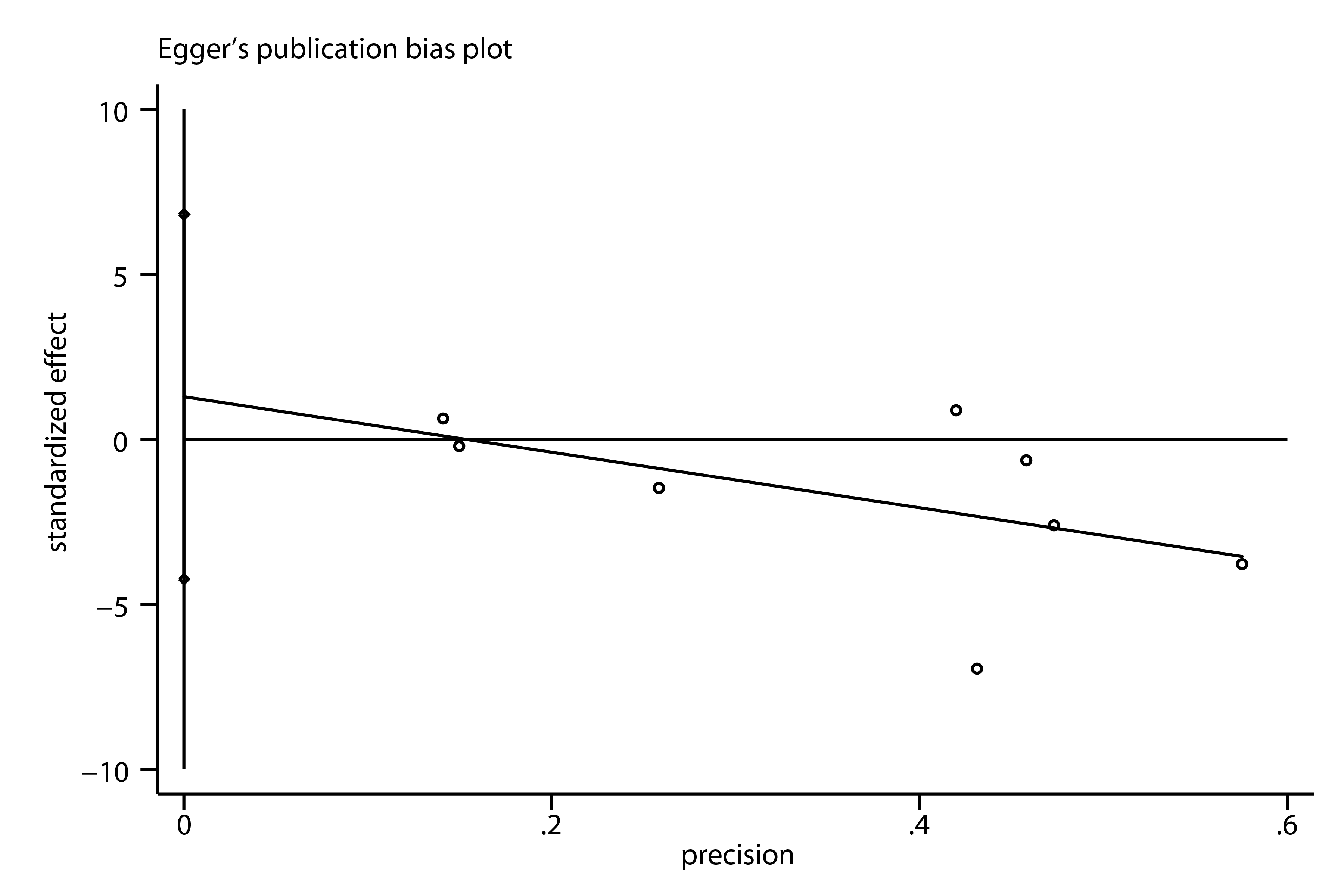
**

**Fig S19. Forest plot of serum potassium level among patients with hemodialysis vs peritoneal dialysis**

**
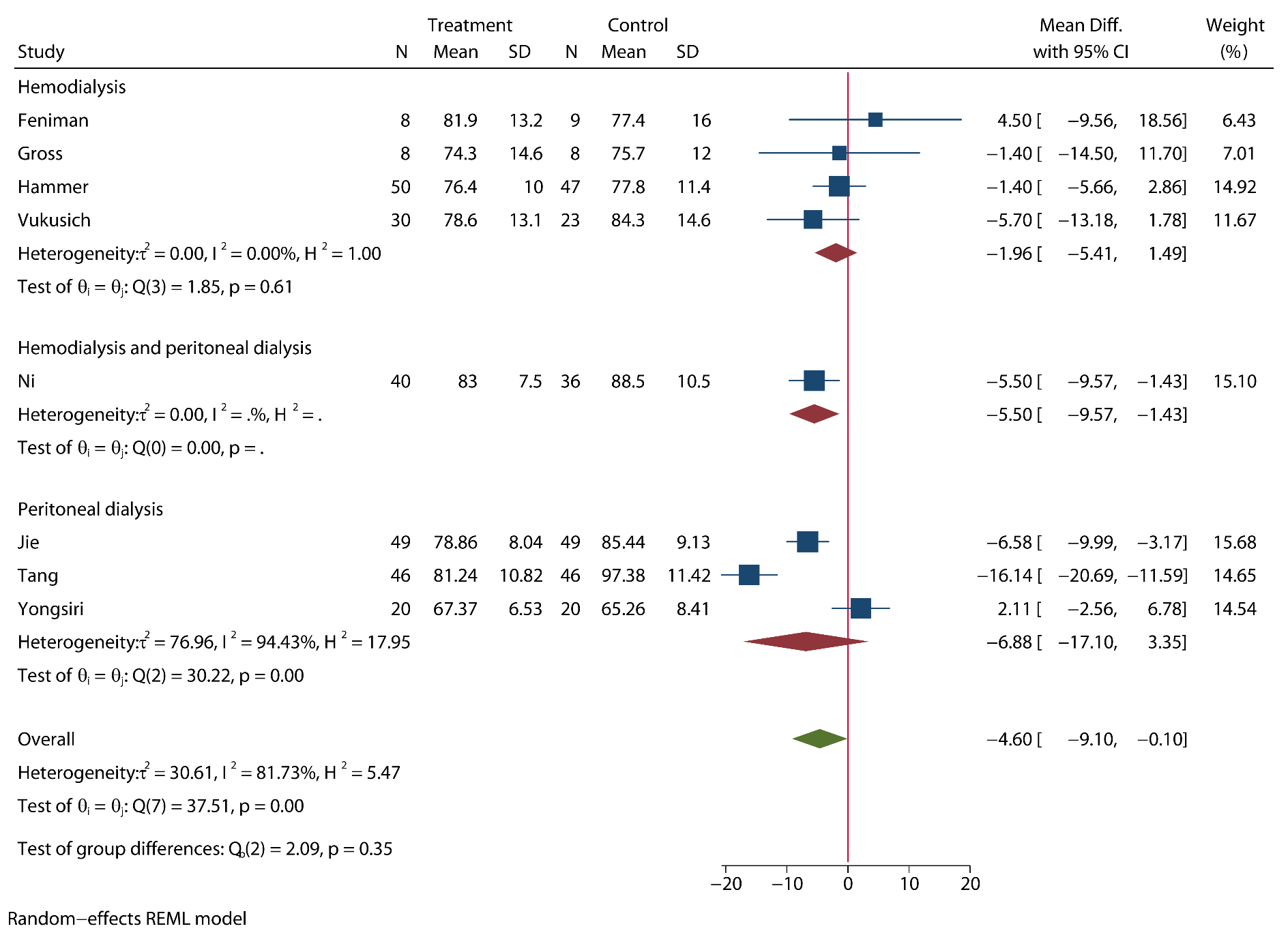
**

**Fig S20. Forest plot of serum potassium level.**


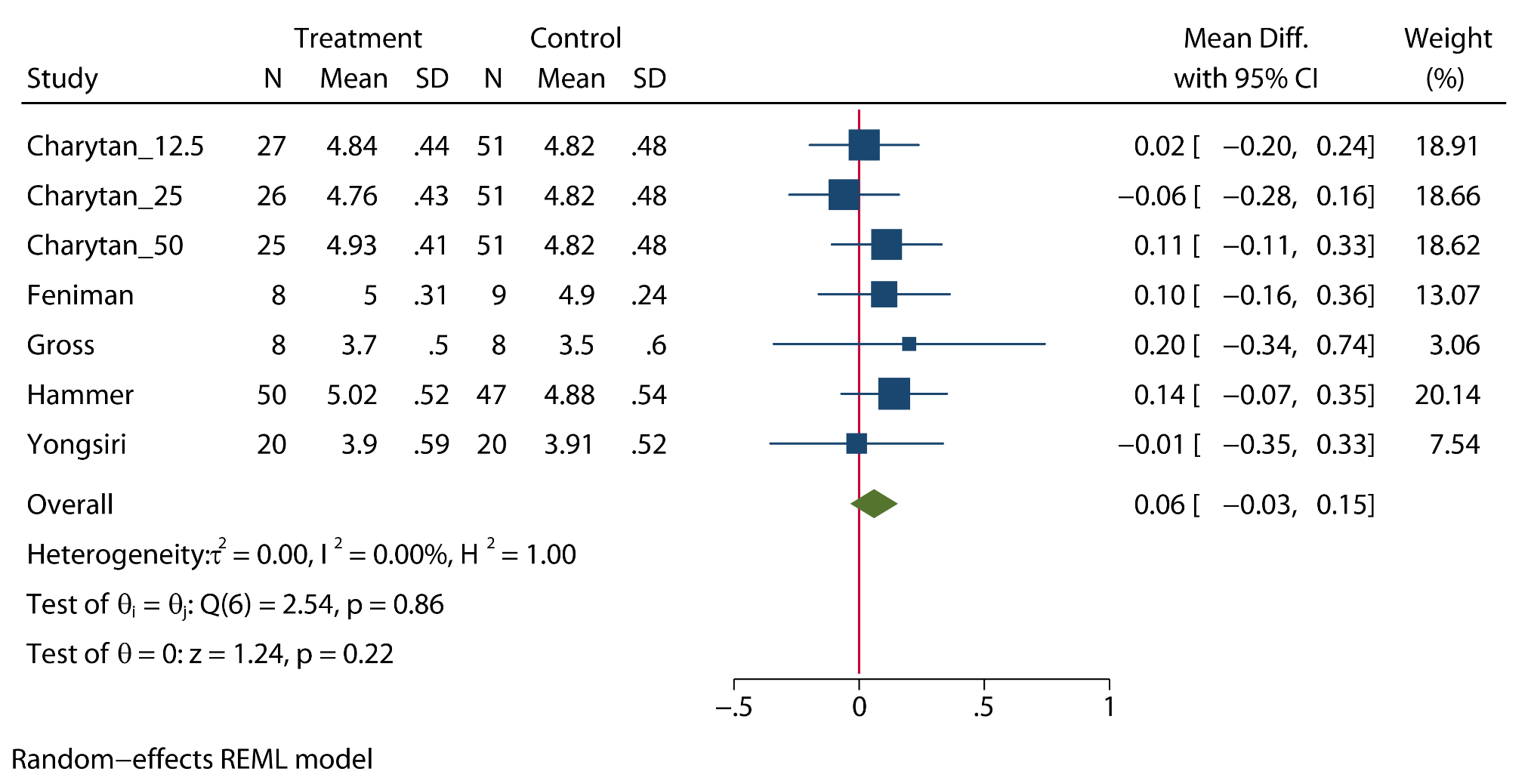


**Table S1. Main characteristics of the included studies.**

| **Study** | **Area** | **Design** | **Sample Size** | |  | **Age** | **Dialysis**  **solution** | **Journal** | **Intervention** | | **Follow-up**  **(Months)** | **Main results** |
| --- | --- | --- | --- | --- | --- | --- | --- | --- | --- | --- | --- | --- |
|  |  |  | **T(F/M)** | **C(F/M)** |  | **MRAs/ control** |  |  | **MRA** | **control** |  |  |
| Charytan et al.  2018a | USA | Parallel | 27(12/15) | 51(19/32) |  | 56.8±11.5/55.1±13.6 | HD | Kidney Int | Spironolactone  12.5/25/50 mg qd | Placebo | 9 | LVMI, SP, ACM  LVEF, CCVM |
| Charytan et al.  2018b([Charytan et al., 2019](#_ENREF_5)) | USA | Parallel | 26(7/19) | 51(19/32) |  | 56.8±11.5/53.3±13.5 | HD | Kidney Int | Spironolactone  12.5/25/50 mg qd | Placebo | 9 | LVMI, SP, ACM  LVEF, CCVM |
| Charytan et al.  2018c([Charytan et al., 2019](#_ENREF_5)) | USA | Parallel | 25(6/19) | 51(19/32) |  | 56.8±11.5/55.5±9.8 | HD | Kidney Int | Spironolactone  12.5/25/50 mg qd | Placebo | 9 | LVMI, SP, ACM  LVEF, CCVM |
| Feniman et al.  2015([Feniman-De-Stefano et al., 2015](#_ENREF_7)) | Brazil | Parallel | 8(4/4) | 9(5/4) |  | 52 ± 19.2/ 56 ± 10.9 | HD | Ther Adv Cardiovasc Dis | Spironolactone 12.5 mg daily  for 1 weeks, then 25 mg daily | Placebo | 6 | SP, LVMI, LVEF, BP |
| Gross et al.  2005([Gross, Rothstein, Dombek & Juknis, 2005](#_ENREF_10)) | USA | Crossover | 8(5/3) | 8(5/3) |  | 53 ± 10 | HD | Am J Kidney Dis | Spironolactone  50 mg bid | Placebo | 0.5 | BP, SP |
| Hammer et al.  2019([Hammer et al., 2019](#_ENREF_11)) | Germany | Parallel | 50(10/40) | 47(12/35) |  | 59.9 ± 13.4/ 60.6 ± 13.1 | HD | Kidney Int | Spironolactone  50 mg daily | Placebo | 40 | LVMI, BP,  LVEF, SP |
| Ito et al.  2014([Ito et al., 2014](#_ENREF_14)) | USA | Parallel | 78(23/55) | 80(22/58) |  | 57.4 ± 12.3/ 55.6 ± 14.4 | PD | J Am Soc Nephrol | Spironolactone  25 mg daily | None | 6 | ACM |
| Jie et al.  2019([Jie, Wang, Zhao & Dong, 2019](#_ENREF_15)) | China | Parallel | 49(21/28) | 49(19/30) |  | 58.6 ± 3.2/ 57.8 ± 3.5 | HD | Med J Chin PAP | Spironolactone  20 mg daily | Placebo | 3 | BP, |
| Lin et al.  2015([Lin, Zhang, Zhang & Lin, 2016](#_ENREF_19)) | China | Parallel | 125  (52/73) | 128  (48/80) |  | 57.4 ± 12.3/ 55.6 ± 14.4 | HD and PD | J Clin Hypertens | Spironolactone  25 mg daily | Placebo | 24 | ACM |
| Matsumoto  et al.2014([Matsumoto et al., 2014](#_ENREF_20)) | Japan | Parallel | 157  (44/113) | 152  (62/90) |  | 67.4 ± 12.3/ 67.7 ± 11.2 | HD | J Am Coll Cardiol | Spironolactone  25 mg daily | None | 36 | CCVM ACM |
| Ni et al.  2014([Ni et al., 2014](#_ENREF_23)) | China | Parallel | 40(16/24) | 36(15/21) |  | 55.7 ± 12.3/ 54.9 ± 14.2 | HD and PD | J Clin Hypertens | Spironolactone  25 mg daily | Placebo | 12 | BP |
| Tang et al.  2019([Tang, 2019](#_ENREF_40)) | China | Parallel | 46(25/21) | 46(22/24) |  | 54.9 ± 3.2/ 55 ± 3.1 | HD | The Med Forum | Spironolactone  20 mg daily | Placebo | 6 | BP |
| Taheri et al.  2009([Taheri et al., 2009](#_ENREF_39)) | Iran | Parallel | 8(3/5) | 8(2/6) |  | 59.5 ± 6.5/ 56.8 ± 9.3 | HD | Saudi J Kidney Dis Transpl | Spironolactone  25 mg tiw | Placebo | 6 | CCVM, ACM |
| Taheri et al.  2012([Taheri, Mortazavi, Pourmoghadas, Seyrafian, Alipour & Karimi, 2012](#_ENREF_38)) | Iran | Parallel | 9(4/5) | 9(4/5) |  | 50.7 ± 17.4/ 57.2 ± 13.1 | PD | Saudi J Kidney Dis Transpl | Spironolactone  25 mg daily | Placebo | 6 | CCVM, ACM |
| Vukusich et al.  2010([Vukusich et al., 2010](#_ENREF_43)) | Chile | Parallel | 30(10/20) | 23(9/14) |  | 55.6 ± 3.6/ 60.1 ± 5.2 | HD | Clin J Am Soc Nephrol | Spironolactone  50 mg tiw | Placebo | 24 | CIMT, BP, ACM |
| Wang et al.  2018([Wang, LIN, Zhao & Cai, 2018](#_ENREF_45)) | China | Parallel | 48(21/27) | 48(19/29) |  | 52.6 ± 6.7/ 53.3 ± 7.2 | PD | Chin Gen Pract | Spironolactone  20 mg daily | Placebo | 12 | LVMI |
| Walsh et al.  2015([Walsh et al., 2015](#_ENREF_44)) | USA | Parallel | 77(30/47) | 77(28/49) |  | 62.1 ± 14.6/ 63.1 ± 13.7 | HD | J Am Soc Nephrol | Eplerenone  50 mg daily | Placebo | 3 | ACM |
| Yongsiri et al.  2014([Yongsiri et al., 2015](#_ENREF_47)) | Thailand | Crossover | 20(12/8) | 20(12/8) |  | 52.42 ± 12.35 | PD | Ther Apher Dial | Spironolactone  25 mg daily | Placebo | 4 | BP, SP |

Abbreviations: ACM, all-cause mortality; CVM, cardiovascular mortality; HD: Hemodialysis; LVEF: left ventricular ejection fraction; LVMI: left ventricular mass index; PD; Peritoneal Dialysis; SP: serum potassium.

**Table S2. Summary results of MRAs for the cardiovascular effects in dialysis patients.**

N/Pt-yr: No. of studies/Patient-years

| Point of interest | | | |  | Effects Model | | |  | Heterogeneity | | |
| --- | --- | --- | --- | --- | --- | --- | --- | --- | --- | --- | --- |
| Parameter | subgroup | | N/Pt-yr |  | Pooled Estimate | 95% confidence interval | P-value |  | Chi^2^ | I^2^ (%) | P-value |
| All-cause mortality | | HD | 7/728 |  | 0.52 | 0.23 to 1.22 |  |  | 0.30 | 23.46 | 0.35 |
|  | | HD and PD | 1/253 |  | 0.44 | 0.21 to 0.92 |  |  | NA | NA | NA |
|  | | PD | 2/224 |  | 0.38 | 0.09 to 1.67 |  |  | 0.00 | 0.00 | 0.91 |
|  | | Spironolactone | 9/1051 |  | 0.42 | 0.27 to 0.66 |  |  | 0.00 | 0.00 | 0.57 |
|  | | Eplerenone | 1/154 |  | 0.49 | 0.04 to 5.56 |  |  | NA | NA | NA |
| Cardiovascular mortality | | HD | 6/710 |  | 0.73 | 0.25 to 2.09 | 0.50 |  | 0.44 | 25.16 | 0.29 |
|  | | HD and PD | 1/253 |  | 0.35 | 0.16 to 0.80 | 0.01 |  | NA | NA | NA |
|  | | PD | 2/176 |  | 0.18 | 0.03 to 1.10 | 0.06 |  | 0.00 | 0.00 | 0.64 |
|  | | Spironolactone | 8/985 |  | 0.43 | 0.24 to 0.75 | 0.02 |  | 0.00 | 0.00 | 0.31 |
|  | | Eplerenone | 1/154 |  | 0.49 | 0.04 to 5.56 | 0.57 |  | NA | NA | NA |
| Serum potassium | | HD | 4/183 |  | -1.96 | -5.41 to 1.49 | 0.28 |  | 0.00 | 0.00 | 0.61 |
|  | | HD and PD | 1/76 |  | -5.50 | -9.57 to -1.43 | 0.04 |  | NA | NA | NA |
|  | | PD | 3/230 |  | -6.88 | -17.1 to 3.35 | 0.15 |  | 76.96 | 94.43 | <0.01 |
| LVEF | | NA | 5/345 |  | -2.71 | -4.86 to -0.56 | 0.01 |  | 2.75 | 47.90 | 0.10 |
| LVMI | | NA | 9/441 |  | 8.58 | 3.69 to 13.46 | <0.01 |  | 14.46 | 40.87 | 0.15 |
| SBP | | NA | 8/489 |  | -7.40 | -10.6 to -4.2 | <0.01 |  | 5.53 | 27.29 | 0.18 |
| DBP | | NA | 8/489 |  | -4.60 | -9.10 to -0.10 | 0.04 |  | 30.61 | 81.73 | <0.01 |

**Table 3. Search strategy**

| **Database** | **No** | **PICOS** | **Strategy** |
| --- | --- | --- | --- |
| PubMed | #1 | P | Renal Dialysis [MeSH] OR Dialysis OR Hemodialysis OR Haemodialysis OR ESRD OR Hemodialyses OR Peritoneal Dialysis |
|  | #2 | I | Mineralocorticoid receptor antagonists [MeSH] OR Mineralocorticoid Antagonists OR Aldosterone Receptor Antagonist OR Mineralocorticoid Antagonist OR Aldosterone Antagonist |
|  | #3 | C | NA |
|  | #4 | O | Cardiovascular Diseases[MeSH] OR Hyperkalemia [MeSH] OR Blood Pressure [MeSH] OR heart OR cardiovascular OR myocardial OR stroke OR hypertension OR coronary heart disease OR coronary heart diseases OR Hyperkalemias OR Hyperpotassemia OR Hyperpotassemias OR Serum Potassium OR Diastolic Pressure OR Pulse Pressure OR Systolic Pressure OR Left Ventricular Mass Index OR Left Ventricular Ejection Fraction OR All-cause Mortality |
|  | #5 | S | (randomized controlled trial[Publication Type] OR randomized[TIAB] OR randomised[TIAB] OR placebo[TIAB])) NOT (Review[Publication Type]) NOT (meta-analysis[Publication Type]) NOT (Comment[Publication Type]) NOT (Letter[Publication Type]) |
|  | #6 = #1AND#2 AND#4 AND #5 | NA | ((((Renal Dialysis [MeSH] OR Dialysis OR Hemodialysis OR Haemodialysis OR ESRD OR Hemodialyses OR Peritoneal Dialysis)) AND (Mineralocorticoid receptor antagonists [MeSH] OR Mineralocorticoid Antagonists OR Aldosterone Receptor Antagonist OR Mineralocorticoid Antagonist OR Aldosterone Antagonist)) AND ((randomized controlled trial[Publication Type] OR randomized[TIAB] OR randomised[TIAB] OR placebo[TIAB])) NOT (Review[Publication Type]) NOT (meta-analysis[Publication Type]) NOT (Comment[Publication Type]) NOT (Letter[Publication Type])) |
| Embase | #7 | P | 'Dialysis'/exp OR 'hemodialysis':ab,ti OR 'ESRD':ab,ti OR ‘Haemodialysis’:ab,ti OR ‘Renal Dialysis’:ab,ti OR 'Renal Dialysis':ab,ti OR ' Peritoneal Dialysis ':ab,ti |
|  | #8 | I | 'mineralocorticoid antagonist'/exp OR 'aldosterone antagonist':ab,ti OR 'hormone antagonist':ab,ti OR 'steroid hormone antagonist':ab,ti OR 'corticosteroid antagonist':ab,ti |
|  | #9 | C | NA |
|  | #10 | O | 'cardiovascular disease'/exp OR 'Hyperkalemia'/exp OR 'Blood Pressure'/exp OR 'heart':ab,ti OR 'cardiovascular':ab,ti OR 'myocardial':ab,ti OR 'stroke':ab,ti OR 'hypertension':ab,ti OR 'coronary':ab,ti OR 'Hyperkalemias':ab,ti OR 'Hyperpotassemia':ab,ti OR 'Hyperpotassemias':ab,ti OR 'Serum Potassium':ab,ti OR ‘Diastolic Pressure’:ab,ti OR ‘Pulse Pressure’:ab,ti OR ‘Systolic Pressure’:ab,ti OR ‘Kidney Diseases’:ab,ti OR ‘Kidney Disease’:ab,ti OR ‘Retinal Disease’:ab,ti OR ‘Left Ventricular Mass Index’:ab,ti OR ‘Left Ventricular Ejection Fraction’:ab,ti OR ‘All-cause Mortality’:ab,ti |
|  | #11 | S | 'randomized controlled trial'/exp NOT review:it |
|  | #12 = #7 AND #8 AND #10 AND #11 | NA | ('Dialysis'/exp OR 'hemodialysis':ab,ti OR 'ESRD':ab,ti OR ‘Haemodialysis’:ab,ti OR ‘Renal Dialysis’:ab,ti OR 'Renal Dialysis':ab,ti OR ' Peritoneal Dialysis ':ab,ti) AND ('mineralocorticoid antagonist'/exp OR 'aldosterone antagonist':ab,ti OR 'hormone antagonist':ab,ti OR 'steroid hormone antagonist':ab,ti OR 'corticosteroid antagonist':ab,ti) AND ('cardiovascular disease'/exp OR 'Hyperkalemia'/exp OR 'Blood Pressure'/exp OR 'heart':ab,ti OR 'cardiovascular':ab,ti OR 'myocardial':ab,ti OR 'stroke':ab,ti OR 'hypertension':ab,ti OR 'coronary':ab,ti OR 'Hyperkalemias':ab,ti OR 'Hyperpotassemia':ab,ti OR 'Hyperpotassemias':ab,ti OR 'Serum Potassium':ab,ti OR ‘Diastolic Pressure’:ab,ti OR ‘Pulse Pressure’:ab,ti OR ‘Systolic Pressure’:ab,ti OR ‘Kidney Diseases’:ab,ti OR ‘Kidney Disease’:ab,ti OR ‘Retinal Disease’:ab,ti OR ‘Left Ventricular Mass Index’:ab,ti OR ‘Left Ventricular Ejection Fraction’:ab,ti OR ‘All-cause Mortality’:ab,ti) AND ('randomized controlled trial'/exp NOT review:it) |

Abbreviation: NA, not applicable

**Table 4. Inclusion/exclusion criteria of literature**

| **PICOS** | **Inclusion** | **Exclusion** |
| --- | --- | --- |
| P | Adults participants (≥18 y) were on dialysis for at least 1 month, irrespective of age, gender, and race. | Children, pregnant women, and patients with a history of kidney transplantation were excluded |
| I | Participants were on hemodialysis or peritoneal dialysis for at least 1 month, and oral MRAs(spironolactone or eplerenone) for at least 2 weeks. And patients with a history of kidney transplantation were excluded. | Not available |
| C | No intervention, or with placebo or a lifestyle intervention. | Not available |
| O | One of the following outcomes must have been included: serum potassium (SP), left ventricular mass index (LVMI), left ventricular ejection fraction (LVEF), cardiovascular and cerebrovascular mortality (CCVM), all-cause mortality (ACM), systolic blood pressure (SBP) or diastolic blood pressure (DBP). | Not available |
| S | RCT irrespective of blinding or arm | 1) Articles without peer reviewed or unpublished  2) Studies that were repeatedly published or had qualitative outcomes  3) Quasi-experimental studies and observational studies |
